# Endophytic *Aspergillus fumigatus* modulates growth, oxidative stress, and cadmium accumulation in okra under salinity and heavy-metal stress

**DOI:** 10.1371/journal.pone.0356384

**Published:** 2026-08-13

**Authors:** Sobia Khan, Salman Khan, Afshan Afshan, Muhammad Arif, Humaira Gul, Muhammad Hamayun, Mamoona Rauf, Douglas Law, Bandar M. Almunqedhi, Mohamed A. El-Tayeb, Ahmed Othman Alsabih, Waheed Ahmad, Patricio R. De los Ríos-Escalante, Ayaz Ahmad

**Affiliations:** 1 Department of Biotechnology, Abdul Wali Khan University Mardan, Khyber Pakhtunkhwa, Pakistan; 2 Department of Botany, Abdul Wali Khan University Mardan, Khyber Pakhtunkhwa, Pakistan; 3 Faculty of Health and Life Sciences, INTI International University, Nilai, Negeri Sembilan, Malaysia; 4 Botany and Microbiology Department, College of Science, King Saud University, Saudi Arabia; 5 Department of Physiology, College of Medicine, King Saud University, Riyadh, Saudi Arabia; 6 Minnesota Dental Research Center for Biomaterials and Biomechanics, School of Dentistry, University of Minnesota, Minneapolis, Minnesota, United States of America; 7 Núcleo de Estudios Ambientales, Facultad de Recursos Naturales, Universidad Católica de Temuco, Chile; 8 Departamento de Ciencias Biológicas y Químicas, Facultad de Recursos Naturales, Universidad Católica de Temuco, Chile; Yeungnam University, KOREA, REPUBLIC OF

## Abstract

Abiotic stress factors, such as salinity and heavy metals (HM), significantly reduce okra (*Abelmoschus esculentus* L.) yield, an important vegetable crop cultivated globally. The symbiotic relationship between endophytic fungi and plants enhances plant growth and helps plants overcome such stresses. This study was designed to isolate and examine endophytic fungi from *Ziziphus lotus* roots for their growth-promoting potential under salt (250 mM NaCl) and cadmium (100 ppm Cd) stress. The growth of okra was significantly enhanced by inoculation with *Aspergillus fumigatus* (SOA), resulting in a 28.9% increase in plant height (33.24 cm vs. 25.79 cm in the control) and improved biomass, chlorophyll content, and phytohormone regulation. Chlorophyll A content rose by 108.4% in SOA-treated plants (4.894 mg/g FW) relative to control (2.348 mg/g FW), while shoot dry weight increased more than threefold (4.365 g vs. 1.42 g in control). Abscisic acid (ABA) content decreased in SOA-treated plants, indicating a reduced stress response, whereas enhanced gibberellic acid (GA) and indole-3-acetic acid (IAA) content promoted growth. Biochemical analysis revealed higher accumulation of lipids, sugars, phenols, and flavonoids, resulting in improved stress adaptation. Oxidative damage was reduced through high antioxidant enzyme activities (CAT and POD). SOA modulated cadmium uptake, thereby reducing heavy metal toxicity. These findings showed that endophytic fungi have the potential to enhance plants’ resilience and provide a promising controlled-environment approach to enhance crop productivity in metal- and salt-contaminated soils.

## 1. Introduction

In all parts of the world, saline conditions cause severe losses in plant growth and productivity because of their negative effects on biochemical, molecular, and physiological processes. Plant growth reduction is triggered by several factors, including osmotic stress, ionic toxicity, ROS production, hormonal imbalance, and reduced nutrient mobilization [1]. Crop productivity is adversely affected by waterlogging, drought, high and low temperature stress, salinity, soil pH, pesticides, and other environmental stresses, all of which are major limiting factors in crop production [2]. Among these, soil and water salinity remain one of the most critical problems worldwide, leading to persistent reductions in crop productivity and arable land. Globally, salinity affects 33% of irrigated land and 20% of cultivated land [3]. Due to saline water irrigation, poor agricultural practices, and rock weathering, soil salinization is increasing by nearly 10% per year [4].

Abiotic stresses are among the major limitations to crop productivity [5,6]. Close to 90% of arable land is susceptible to one or more abiotic stress factors, which can reduce yield by up to 70% in staple crops. Integrated projections based on climate models and crop productivity simulations indicate a continued decline in yields of major crops such as rice, wheat, and maize, posing a serious threat to global food security [7]. Different environmental stresses, such as drought [8] and HM contamination [9], interfere with vital physiological processes, including photosynthesis, respiration, nutrient transport, ion uptake, transpiration, stomatal regulation, seed germination, and water balance. Soil salinity in irrigated areas has increased by 37% over the last 20 years [10]. Moreover, changing precipitation patterns and increased evapotranspiration due to global warming have amplified the occurrence and severity of drought events. A recent meta-analysis predicted that global average temperatures could increase by 2.0–4.9 °C by the late 21^st^ century [11]. Extreme heat and dryness represent an additional threat to agricultural croplands already susceptible to salinity, particularly in arid and semi-arid regions [12]. Furthermore, excessive production of reactive oxygen species (ROS) triggered by NaCl stress damages important biomolecules such as proteins, lipids, and DNA, thereby disturbing cellular redox equilibrium [13].

Photosynthesis, respiration, ion transport, transpiration, stomatal behavior, seed germination, mineral uptake, and water relations are among the most important plant physiological processes that are severely affected by abiotic stresses such as salinity [14] and HM contamination [15]. Under NaCl stress, plants produce high levels of phytohormones, such as ABA, which are upregulated by genes involved in NaCl and osmotic-stress tolerance [16]. Similarly, JA plays a key role in defensive responses to wounding and pathogen attacks [17]. SA is a phytohormone that regulates plants’ defense system against pathogenic attack and plays a role in alleviating abiotic stress [18]. Cadmium (Cd) accumulation in agricultural soil is a major environmental problem [19,20]. The toxic, persistent, and bioaccumulative nature of Cd poses a serious risk to agricultural productivity and food chain security [21]. Similarly, in okra (*Abelmoschus esculentus*), Cd present in the growth substrate inhibits carbon fixation, thereby decreasing photosynthetic rate and, hence, pod yield [22]. Antioxidant enzymes, which play an important role in protecting cells from damage, are significantly changed in okra after exposure to metal [22]. Increased concentrations of hydrogen peroxide and MDA, lower stomatal conductance, leaf relative water content, and transpiration are among the signs of Cd-induced oxidative stress in okra seedlings [23]. Apart from these biochemical disturbances, Cd accumulation in okra also causes nutritional imbalances, membrane damage, and poor growth, resulting in structural and physiological deformities and low yield [22].

The symbiotic association between endophytic fungi and host plants has attracted increasing attention as a low-cost, eco-friendly strategy for improving crop stress tolerance. Plant growth-promoting endophytic fungi (PGPEF) act as natural biocontrol and biofertilization agents, helping host plants adapt to adverse environmental conditions [24]. Several *Aspergillus* species have demonstrated this potential: endophytic *Aspergillus terreus* and *Aspergillus niger* isolates have alleviated NaCl stress and oxidative damage in *Vigna radiata* [25,26]. *Aspergillus fumigatus* has been shown to promote plant growth and modulate host phytohormone levels by producing gibberellin [27]. Endophytic *Aspergillus* strains have reduced Cd and chromium accumulation while improving growth and antioxidant status in okra and tomato [28,29]. However, these studies have generally examined salinity or heavy-metal stress in isolation, and reports specifically addressing okra’s NaCl tolerance or Cd response have likewise considered each stress separately rather than in combination.

Although previous research has explored the potential of endophytic fungi to enhance plant stress tolerance, the role of endophytic *Aspergillus fumigatus* under combined stress from NaCl and Cd remains unclear. To the best of our knowledge, no study has been conducted to date on the efficacy of an endophytic *A. fumigatus* strain in promoting growth, regulating the antioxidant defense system, and reducing Cd concentration in okra plants *(Abelmoschus esculentus* L.) under combined NaCl and Cd stresses. To address this knowledge gap, the present study isolates an endophytic strain, *Aspergillus fumigatus*, from the roots of *Ziziphus lotus* and explores its potential to improve growth, alleviate oxidative damage, and mitigate Cd uptake in okra subjected to combined NaCl and Cd stress. The novelty of this study lies in its four aspects: (i) the fungal strain—a novel *A. fumigatus* isolated endophytic strain not studied before for both abiotic stress tolerance and remediation; (ii) the host plant—a novel okra, which is economically important and accumulates Cd but whose performance under the effect of simultaneous presence of both the stresses has not been studied yet; (iii) the stress treatment—application of the two stresses together rather than separately, which simulates real field situations of plants suffering from the stresses; and (iv) the methodology—multivariate approach used to assess the performance of the plants.

## 2. Methodology

### 2.1 Isolation of endophytic fungi from Z*iziphus lotus roots*

The plant growth-promoting endophytic fungus was isolated from the roots of Z*iziphus lotus* (a common fruit tree of Pakistan). Plant root samples were collected from District Mardan of Khyber Pakhtunkhwa, Pakistan, with permission from the Plant Breeders Rights Registry (PBRR), Government of Pakistan, in accordance with the relevant collection guidelines. The collection and use of the plant material complied with applicable institutional, national, and international regulations. This study was approved by the Graduate Study Committee (GSC) of the Department of Biotechnology, the Sub-ASRB (Advanced Study and Research Board of the Faculty of Chemical and Life Sciences, and the Institutional Animal Care and Ethical Committee of the Department of Biotechnology, Abdul Wali Khan University Mardan (AWKUM), Pakistan. Prof. Dr. Waheed Murad identified the plant material at the Department of Botany, AWKUM, Pakistan. A voucher specimen of *Ziziphus lotus* was deposited in the herbarium of the Department of Botany, AWKUM, Pakistan, under voucher number File/No/AWKUM/BOTANY/HERBARIUM/2024/3065. Roots were washed and treated with 70% ethanol for 1 minute, 2% sodium hypochlorite (NaOCl) for 2 minutes, and then thoroughly washed with autoclaved distilled water. The roots were cut into small pieces with a sterile razor blade and incubated on Hagem minimal medium at 25 °C for seven days. Colonies emerging from the root sections were re-cultured on potato dextrose agar (PDA) medium at 28 °C for a week. The isolated fungal colonies were cultured in Czapek liquid medium (30 °C, 120 rpm) for 7 days. Following the culturing phase, the biomass and fungal filtrate were isolated on filter paper and preserved for later biochemical and metabolic profiling, as documented earlier [30].

### 2.2 Microscopic phenotyping of (*Aspergillus fumigatus*)

The morphological characteristics of isolates were analyzed under a light microscope [31] at 40× and 100 × magnifications, following standard light microscopy procedures. The fungus was stained with staining solution (Lactophenol cotton blue) for clear visibility, as previously reported [32].

### 2.3 Metabolic analysis of fungal endophytic under NaCl and Cd stresses

To evaluate the stress-tolerance potential of the fungal endophytes, selected fungal strains were inoculated into Czapek liquid medium (100 mL), supplemented with different concentrations of NaCl (100, 200, and 300 mM NaCl) and 100 ppm Cd. The flasks were incubated at 30 °C for 7 days in a shaking incubator at 120 rpm, and the growth was tracked. Following the NaCl and Cd tolerance test, all tolerant strains were retained, and the best-performing isolate (SOA) was selected for further studies.

To promote host plant growth, the endophytic fungi produce a range of metabolites. In this research, fungal culture was analyzed for different plant hormones (IAA, GA, ABA, and SA), primary metabolites (proteins, sugars, and lipids), secondary metabolites (phenolics, flavonoids, and proline), and enzymatic and non-enzymatic antioxidants. For plant inoculation, a spore suspension at a final concentration of about 5 × 10^7^ spores/mL was prepared. Biomass and culture supernatant were partitioned with sterilized Whatman filter paper. Culture was centrifuged in a Sartorius Model 2–16 PK centrifuge to obtain the pellet, which was later used to estimate the aforementioned metabolites. The resulting supernatants were kept at −80 °C for subsequent analysis. Catalase (CAT) activity, which allows hydrogen peroxide (H_2_O_2_) to break down, was quantified as a decrease in the absorbance at 240 nm, representing the rate of decomposition of H_2_O_2_ (M H_2_O_2_ min^-1^) [33].

### 2.4 Identification of SOA

Genomic DNA was purified using the DNeasy Plant Mini Kit (QIAGEN) as described previously [34]. Fungal isolate (SOA) *Aspergillus fumigatus* was identified by sequencing the ITS1-5.8S-ITS2 region of the nuclear DNA, with the specific primers ITS1 (5′-TCCGTAGGTGAACCTGCGG-3′) and ITS4 (5′-GCTGCGTTCTTCATCGATGC-3′), as reported [35]. Homologous sequences were obtained and aligned using the CLUSTAL W algorithm. Phylogenetic and evolutionary inferences were then conducted in MEGA X [36]. The ITS sequence obtained from isolate SOA was deposited in the NCBI GenBank database under accession number ON202840.

### 2.5 Effect of SOA on the okra plant under NaCl and Cd stress

Seeds of an *Abelmoschus esculentus* genotype were obtained from the Plant Genetic Resources Program (PGRP), Pakistan Agricultural Research Council (PARC)/National Agriculture Research Center (NARC), Islamabad, Pakistan, through an available public germplasm source [37]. No field collection of okra seed specimens was undertaken for this study. Physical appearance was used to select healthy, mature, and proportionate seeds. Autoclaved distilled water was used to wash the seeds three times. Sterilization of the okra seeds was accomplished using 70% ethanol. The soil was autoclaved to eliminate indigenous microorganisms, ensuring that the observed effects were solely attributable to the inoculated fungal endophyte. Okra seeds were sown in fungal biomass-amended, autoclaved soil, at 2 g per 100 g of soil after three cold-water washes and subsequent washes with distilled water. The pots were incubated in a controlled growth chamber with 14 hours of light (28 ± 0.3 °C) and 10 hours of darkness (25 ± 0.3 °C), with a relative humidity of 70%. All treatments had six plants (2 each) in three 18.5 cm-long, 12.5 cm-deep containers. After 5 days of seed germination, 5 mL of fungal culture filtrate (FCF) was added to each pot. Cd (100 ppm) and NaCl (250 mM) stresses were applied after 10 days of germination. Tap water was used for watering, and treatment was repeated twice at 7-day intervals until the okra plants were harvested. After 35 days of growth, the experiment was terminated by uprooting the okra plants and separating the roots. Shoot and root biomass were measured. The experiment used a completely randomized design (CRD) with eight treatments and three biological replicates. The design of this experimental setup had a set of measurements, and each replicate consisted of three plants/pots and two technical replicates/plant. For plant sampling, 3 plants were randomly collected from each treatment, carefully cleaned, and preserved in labeled paper bags.

### 2.6 Experimental design

Treatment 1. Control

Treatment 2. SOA

Treatment 3. Cadmium stress (Cd)

Treatment 4. NaCl stress

Treatment 5. Cd + SOA

Treatment 6. NaCl + SOA

Treatment 7. Cd + NaCl + SOA

Treatment 8. Cd + NaCl

### 2.7 Growth parameters

At the conclusion of the experiment, the total yield was determined by measuring shoot and root lengths and the fresh and dry weights of the okra plants.

### 2.8 Chlorophyll and carotenoid contents

The previously described method was used to determine chlorophyll and carotenoid contents [38].

### 2.9 Determination of Endogenous IAA, GA, SA, and ABA

Fresh leaves (0.1 g) were ground in liquid nitrogen to determine endogenous IAA contents. IAA was purified and extracted as described earlier [39]. Gibberellic acid and ABA were quantified as described [40]. Salicylic acid (SA) was estimated using the method reported elsewhere [41].

### 2.10 Determination of secondary metabolites

Total flavonoids were estimated by the AlCl_3_ method as reported [42]. Phenolic content was determined as described earlier [43]. Total soluble sugar was estimated according to the previously reported procedure [44], with OD measured at 485 nm. Total lipids were extracted using the reported method [45].

### 2.11 Determination of antioxidant activities

DPPH (1,1-diphenyl-2-picrylhydroxyl) scavenging activity was determined with minor modifications to the previously reported procedure [46]. 0.1 g of plant material was dissolved in 1 mL of methanol and treated with a 0.004% DPPH solution in methanol. 1 mL of DPPH solution was added to the 0.5 mL sample, and the mixture was incubated at room temperature in the dark for 30 minutes. The DPPH staining was read at 517 nm. Lower absorption indicated improved free radical scavenging, calculated from the formula:


$$\begin{array}{c} \%DPPH=\left(\frac{\text{1}-AE}{AD}\right)\times \text{1}\text{0}\text{0} \end{array}$$

AE = Absorption with extract, AD = Absorption of DPPH solution only.

CAT activity was measured to assess H_2_O_2_ cleavage, following a previously reported method [47]. The reduction in H_2_O_2_ concentration was monitored by the decrease in absorption at 240 nm, which was recorded as the rate of H_2_O_2_ cleavage (M H_2_O_2_ min ^-1^).

Peroxidase activity was evaluated by measuring the dehydrogenation of guaiacol as a substrate, as described earlier [48]. The enzyme was isolated from plant tissue in 3 mL of phosphate buffer (pH 7.0). Preparation of the extract involved grinding 0.1 g of leaves in 1 mL of Tris buffer and then centrifuging at 12,000 rpm for 15 minutes at 5 °C. The supernatant, collected within 2–4 hours, was used as the enzyme source. For carrying out the assay, 3 mL of phosphate buffer (0.1 M), 0.03 mL of H_2_O_2_ (12.3 mM or 0.04%), 0.05 mL of guaiacol solution (20 mM), 0.1 mL of plant extract, and another 0.03 mL of H_2_O_2_ (12.3 mM or 0.04%) were combined in a cuvette. The solution was shaken mildly, and the absorbance was measured at 436 nm.

### 2.12 Reactive Oxygen Species (ROS) accumulation through DAB

To investigate H_2_O_2_ biosynthesis and accumulation, a DAB (3,3′-diaminobenzidine; Sigma, USA) staining assay was performed on leaf discs following the procedure described [49].

### 2.13 Elemental analysis of Cd in plant tissues

As previously described, bioavailability was determined by atomic absorption spectrometry [50]. The plant samples were water-washed, separated into shoots/roots and leaves, and oven-dried for 48 hours at 65 °C until their weight stabilized. The samples were ground to a fine powder using a mortar and pestle. A 0.2 g aliquot of the root/shoot powder was then digested with 5 mL of HNO_3_ (65% w/w) at 110 °C for 2 hours. After cooling, 1 mL of H_2_O_2_ (30% w/w) was added, and the solution was incubated for an hour. The resulting digest was diluted with triple-deionized water in a conical flask [32].

### 2.14 Statistical analyses

All experiments were performed in three biological replicates (six technical replicates per biological replicate), and the results were analyzed using ANOVA in SPSS-20. Statistical differences between mean values were compared using Duncan’s Multiple Range Test (DMRT) at p-value = 0.05 (SPSS Inc., Chicago, IL, USA).

## 3. Results

### 3.1 Morphological and microscopic confirmation of isolated fungal strain

**Fig 1:**
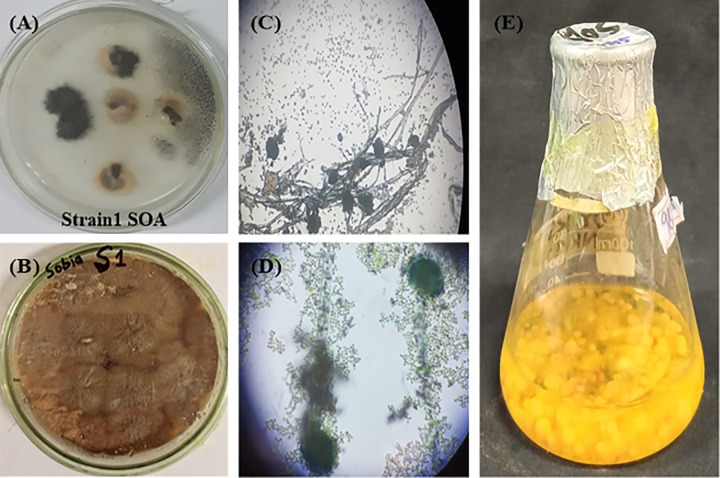
Physiological characteristics and appearance of the SOA strain. (A) Isolated strain from the root section, (B) cultured on a PDA plate, (C, D) microscopic analysis at 40× and 100 × magnification, respectively, and (E) Czapek broth culture of SOA after 7 days of incubation.

The endophytic fungal strain *Aspergillus fumigatus* was isolated from the roots of *Ziziphus lotus* and cultured on both solid and liquid media, specifically Potato Dextrose Agar (PDA) and Czapek broth. The strain exhibited robust growth on solid media, forming compact colonies with a characteristic brownish appearance. Microscopic examination at 40x and 100x magnification revealed clear and well-defined hyphal structures and spore formation, confirming the morphological features of the fungal strain. For primary and secondary metabolite analysis and antioxidant enzyme quantification, the selected endophytic fungal strain was cultured in 100 mL of Czapek broth. The highest biomass yield was recorded as 8.76 g/flask (Fig 1).

### 3.2 Hormonal profiling of SOA under NaCl and Cd stress

**Fig 2:**
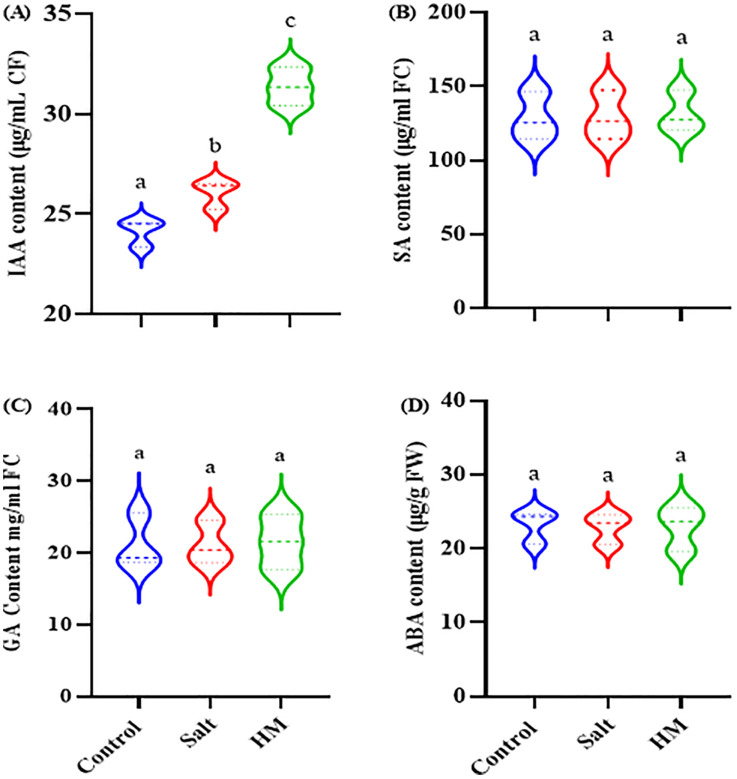
Hormonal profiling of SOA under NaCl and heavy metal stress. (A) Indole-3-acetic acid (IAA) content, (B) Salicylic acid (SA) content, (C) Gibberellic acid (GA) content, and (D) Abscisic acid (ABA) content under control, NaCl, and Cadmium (CD) stress conditions.

The fungal culture filtrate was analyzed for hormonal content under NaCl and Cd stress. Gibberellic acid (GA) production by the endophytic fungus SOA was assessed under three conditions: Control, NaCl stress, and Cd stress. Under control conditions, SOA produced 21 µg/mL of GA. Exposure to NaCl stress increased GA production to 25 µg/mL, while the highest GA levels (28 µg/mL) were observed under Cd stress. Under control conditions, SOA produced 20 µg/mL ABA. NaCl stress increased ABA production to 25 µg/mL, while Cd stress maintained similar levels, with SOA at 25 µg/mL. Also, under control conditions, SOA produced 118.8 µg/mL SA. NaCl stress slightly increased SA production to 124.18 µg/mL, while Cd stress led to the highest levels, with SOA reaching 147.28 µg/mL. Under control conditions, SOA produced 24.1 µg/mL of IAA. NaCl stress increased IAA production to 26.06 µg/mL, while Cd stress led to the highest levels, with SOA reaching 31.37 µg/mL (Fig 2**).**

### 3.3 Antioxidant profiling of SOA under NaCl and Cd stress

**Fig 3:**
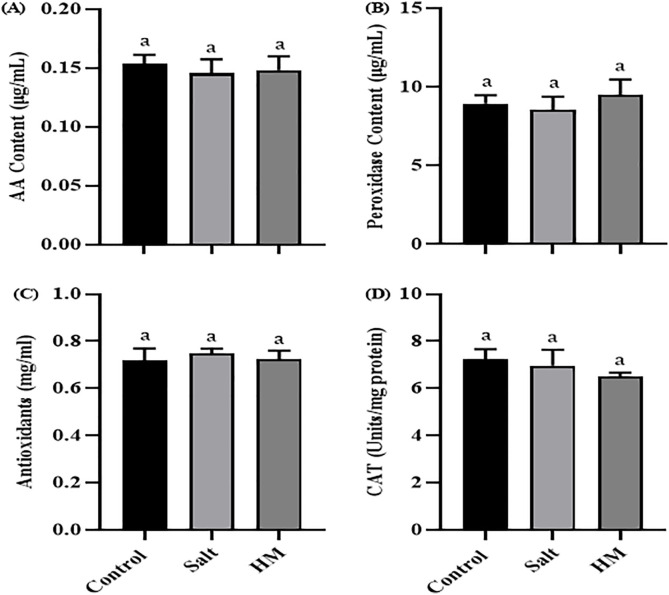
Antioxidant profiling of SOA under NaCl and heavy metal stress. (A) Ascorbic acid (AA) content, (B) Peroxidase activity, (C) Total antioxidant content, and (D) Catalase (CAT) activity under control, NaCl, and Cd stress conditions.

The antioxidant content of SOA was analyzed under control, NaCl, and Cd conditions. Under control conditions, SOA produced 0.6 µg/mL of antioxidants, which increased to 0.75 µg/mL under NaCl stress and peaked at 0.8 µg/mL under Cd stress. AAO production was 0.15 µg/mL under control conditions, decreased to 0.14 µg/mL under NaCl stress, and reached 0.16 µg/mL under Cd stress. CAT activity was 6.2 U/mg protein under control conditions, increased to 7.8 U/mg protein under NaCl stress, and peaked at 8.5 U/mg protein under Cd stress. Peroxide production in SOA was 8.80, 7.65, and 8.55 µg/mL under control conditions, increased to 8.54, 8.65, and 9.36 µg/mL under NaCl stress, and further rose to 9.55, 10.35, and 10.53 µg/mL under Cd stress, as shown in Fig 3.

### 3.4 Effect of NaCl and Cd stress on primary and secondary metabolites

**Fig 4:**
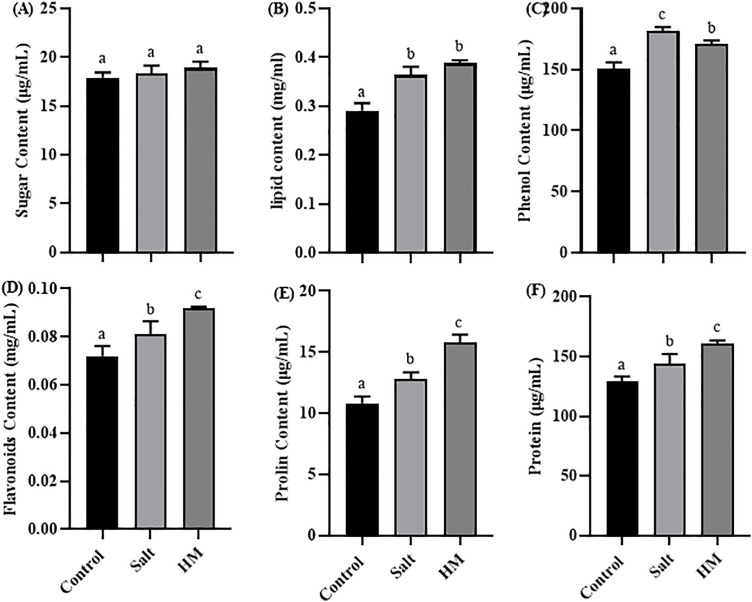
Metabolite content in SOA culture filtrate under control, NaCl, and Cd stress conditions. (A) Sugar content (µg/mL), (B) Lipid content (mg/mL), (C) Phenol content (µg/mL), (D) Flavonoid content (mg/mL), (E) Protein content (µg/mL), and (F) Proline content (µg/mL). All values represent the mean ± standard deviation of three independent replicates.

The primary and secondary metabolite analysis of SOA culture filtrate under control, NaCl, and Cd stress revealed notable variations. Under control conditions, the sugar, lipid, phenol, flavonoid, protein, and proline contents were recorded at **17.94 µg/mL, 291.15 µg/mL, 150.83 µg/mL, 71.92 µg/mL, 128.91 µg/mL, and 10.81 µg/mL**, respectively. NaCl stress significantly enhanced metabolite production, with **sugar (18.80 µg/mL), lipid (364.00 µg/mL), phenol (181.35 µg/mL), flavonoid (81.15 µg/mL), protein (143.54 µg/mL), and proline (12.44 µg/mL)** levels showing noticeable increases. Cd stress further elevated these parameters, peaking at **19.65 µg/mL, 388.12 µg/mL, 171.32 µg/mL, 92.07 µg/mL, 163.43 µg/mL, and 15.53 µg/mL**, respectively (Fig 4).

### 3.5 Molecular identification based on ITS sequences and phylogenetic analysis

**Fig 5:**
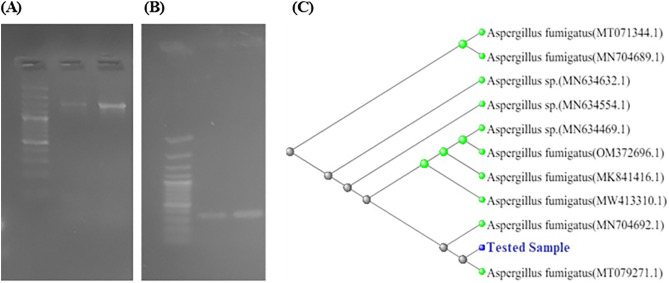
Molecular identification of the SOA endophytic fungal strain. (A) DNA of the isolate with a 1 kb DNA ladder, (B) ITS1-ITS4 region with a 1 kb DNA ladder, and (C) phylogenetic relationship of SOA with closely related species.

For the SOA isolate, the ITS region was amplified and sequenced (Fig 5). Following sequencing, the species or genus of the SOA isolate was identified by aligning the obtained sequence with data in the GenBank database. In Fig 5C, phylogenetic relationships are shown. Sequences used in this study are available online. Following a BLAST search in GenBank, the ITS sequence of SOA was 100% identical to that of *Aspergillus fumigatus* and was classified at the species level. The sequence (accession number ON202840) was deposited in NCBI GenBank.

### 3.6 Effect of NaCl and Cd stress on Okra growth

**Fig 6:**
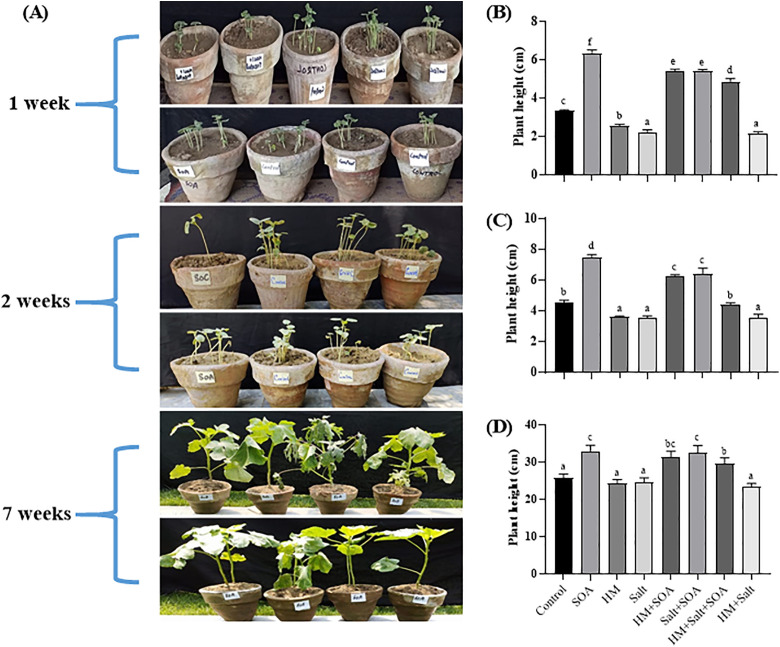
Effect of treatments on plant height under NaCl and Cd stress. (A) Representative plants at weeks 1, 2, 7 after treatment (B) Plant height at week 1: growth was significantly inhibited by NaCl and Cd alone relative to control, while SOA and the SOA-combined treatments (CD + SOA, NaCl + SOA, and CD + NaCl + SOA) showed improved growth. (C) Plant height at week 2: NaCl and CD alone remained suppressed relative to control, with continued improvement in SOA and combined treatments. (D) Plant height at week 7 (final measurement): NaCl and CD alone recovered to control levels, while SOA and SOA-combined treatments showed significantly greater height than control, with CD + NaCl (double stress, no SOA) remaining at the lowest/control level.

Plant height was measured at 1, 2, and 7 weeks under the following treatment conditions: control, SOA, CD, NaCl, and their combinations. Plant height increased significantly under SOA treatment compared to control across all weeks. By week 7, SOA-treated plants (33.24 cm) showed a 28.9% increase over the control (25.79 cm). NaCl and Cd stress reduced growth (24.04 cm and 24.42 cm, respectively), but SOA alleviated these effects, with NaCl + SOA (32.73 cm) and CD + SOA (31.62 cm) showing a marked improvement. The combined CD + NaCl + SOA treatment also enhanced growth (29.36 cm) compared to stressed plants, indicating SOA’s role in mitigating stress-induced growth inhibition (Fig 6).

### 3.7 Chlorophyll content

**Fig 7:**
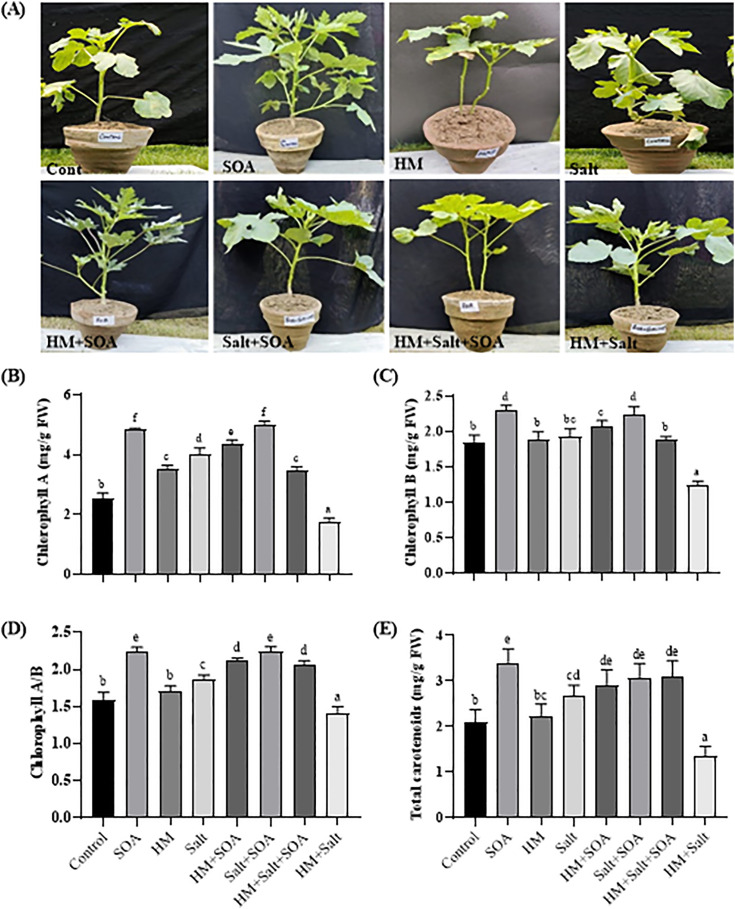
(A) Representative images of plants under different treatments. (B) Chlorophyll A content, (C) Chlorophyll B content, (D) Chlorophyll A/B ratio, and (E) Carotenoid content, highlighting the protective role of SOA against stress-induced reductions.

The chlorophyll and carotenoid content varied significantly across treatments, with SOA showing the most pronounced positive effects. Chlorophyll A content was highest in SOA-treated plants (4.89 mg/g FW), a 108.4% increase over the control (2.35 mg/g FW). NaCl stress significantly reduced chlorophyll A (3.82 mg/g FW), with CD + NaCl showing the lowest levels (1.66 mg/g FW). Similarly, chlorophyll B was highest in NaCl + SOA (2.35 mg/g FW) and lowest in CD + NaCl (1.191 mg/g FW), compared to the control (1.74 mg/g FW). The chlorophyll A/B ratio followed a similar trend, with NaCl + SOA (2.32 mg/g FW) showing the highest value, while CD + NaCl (1.35 mg/g FW) had the lowest. Carotenoid content was highest in NaCl + SOA (3.35 mg/g FW), while CD + NaCl exhibited the lowest levels (1.13 mg/g FW), compared to the control (1.91 mg/g FW). These results indicate that SOA significantly enhanced photosynthetic pigment accumulation, mitigating the adverse effects of NaCl and CD stress (Fig 7).

### 3.8 Effect of SOA on shoot and root fresh and dry weight

**Fig 8:**
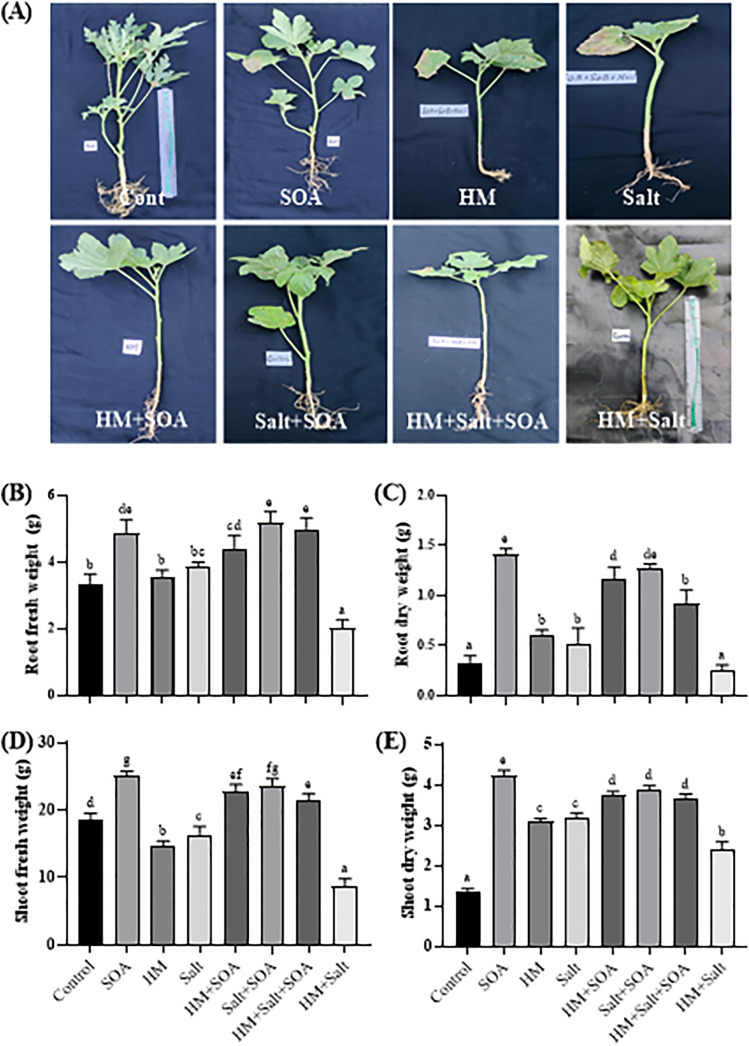
(A) Representative images of whole plant physiology under different treatments, (B) root fresh weight, (C) root dry weight, (D) shoot fresh weight, (E) shoot dry weight.

Shoot fresh and dry weights varied significantly under different treatments. SOA treatment resulted in the highest shoot fresh weight (25.8 g), showing a substantial increase compared to the control (18.5 g). CD and NaCl stress reduced shoot fresh weight to 14.3–15.5 g and 15.1–17.5 g, respectively. However, SOA alleviated stress effects, as CD + SOA (21.9–23.9 g) and NaCl + SOA (22.5–24.6 g) treatments improved shoot fresh weight compared to stressed plants. The CD + NaCl + SOA combination further enhanced biomass (20.8–22.5 g), whereas CD + NaCl alone resulted in the lowest fresh weight (7.6–9.8 g). Similarly, shoot dry weight followed a similar trend. SOA-treated plants exhibited the highest dry weight (4.4 g), a significant increase over the control (1.4 g). CD and NaCl stress reduced dry weight (3.0–3.1 g and 3.1–3.3 g, respectively). However, the application of SOA mitigated these reductions, as CD + SOA (3.6–3.8 g) and NaCl + SOA (3.8–4.0 g) treatments improved dry weight. The CD + NaCl + SOA combination (3.6–3.8 g) also showed positive effects, whereas CD + NaCl treatment resulted in the lowest dry biomass (2.2–2.6 g). SOA significantly increased root fresh (4.5–5.3 g) and dry weight (1.3–1.4 g) compared to the control (3.6–2.9 g and 0.25–0.37 g, respectively). NaCl and CD stress reduced growth, but SOA alleviated these effects, with NaCl + SOA (5.5 g fresh, 1.3 g dry) and CD + SOA (4.7 g fresh, 1.2 g dry) showing improvement. The CD + NaCl + SOA combination further enhanced root biomass, highlighting SOA’s stress-mitigating role (Fig 8).

### 3.9 Phytohormone analysis

**Fig 9:**
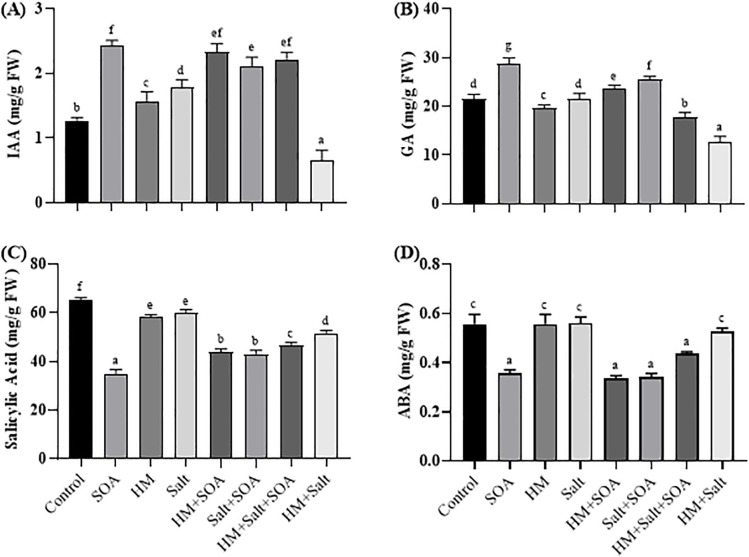
Phytohormonal profiling under different treatments. (A) Indole-3-acetic acid (IAA), (B) Gibberellic acid (GA_3_), (C) Salicylic acid (SA), and (D) Abscisic acid (ABA) contents in plants exposed to control, SOA, CD, NaCl, and combined treatments. SOA application enhanced levels of growth-promoting hormones (IAA and GA_3_) and SA while modulating ABA levels, suggesting a role in stress alleviation and hormonal homeostasis.

The phytohormone analysis revealed significant variations among treatments. Indole-3-acetic acid (IAA) levels peaked in the CD + SOA treatment (3.8 mg/g FW), while the lowest was observed in CD + NaCl (1.0 mg/g FW). Gibberellic acid (GA) showed the highest concentration in SOA (36 mg/g FW) and the lowest in the control (18 mg/g FW). Salicylic acid (SA) was most abundant in the control (4.0 mg/g FW) and least in NaCl + SOA (2.6 mg/g FW). Abscisic acid (ABA) reached its highest level in the control (1.8 mg/g FW) and its lowest in CD + SOA (0.8 mg/g FW) (Fig 9).

### 3.10 Biochemical analysis of secondary metabolites and macromolecules under different treatments

**Fig 10:**
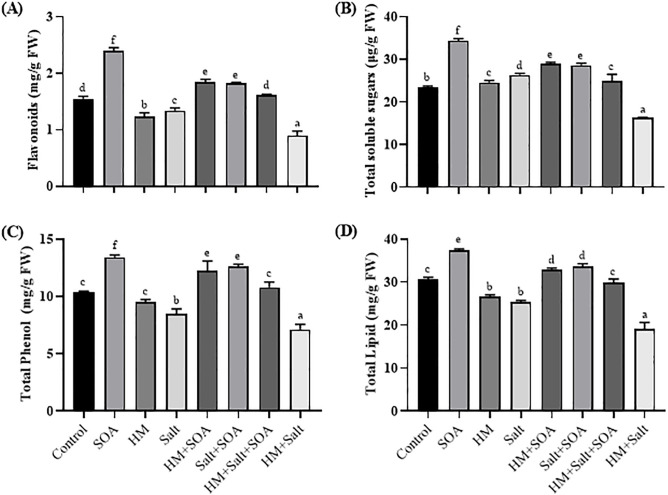
Biochemical characterization of stress markers in plants subjected to different treatments. (A) Flavonoid content, (B) Total soluble sugar, (C) Total phenol content, and (D) Total lipid content. The application of SOA enhanced the production of important metabolites under CD and NaCl stress, reflecting its role in augmenting the biochemical defense response and metabolic stability.

The biochemical evaluation of secondary metabolites and macromolecules subjected to various treatments showed clear-cut variations. The flavonoid content was the highest in SOA (2.8 mg/g FW) and the lowest in CD + NaCl (1.0 mg/g FW). The total soluble sugars were highest in SOA (38 µg/g FW), and the lowest was observed in CD + NaCl (10 µg/g FW). Total phenol content was the highest in SOA (14 mg/g FW) and the lowest in CD (6 mg/g FW). Maxima for total lipids were achieved in NaCl + SOA (380 mg/g FW) and minima for CD + NaCl (140 mg/g FW). These results confirm treatment-specific variations in the accumulation of secondary metabolites and macromolecules (Fig 10).

### 3.11 Antioxidant activity and oxidative stress response

**Fig 11:**
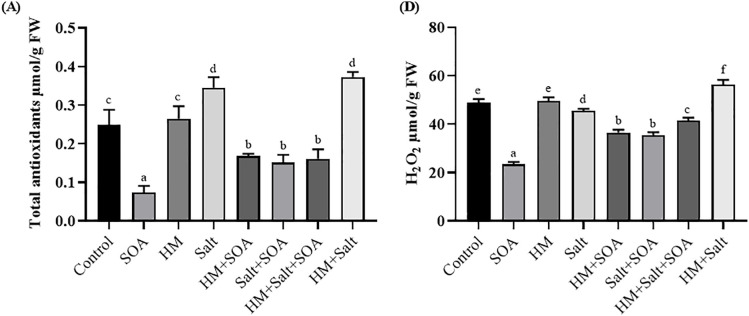
Oxidative stress markers in plants under different treatments. (A) Total antioxidant content, (B) H_2_O_2_ content. SOA, especially under combined CD + NaCl stress, enhanced antioxidant levels and reduced H_2_O_2_ content, indicating improved stress tolerance.

Total antioxidant levels were highest in the *NaCl* and CD + *NaCl* treatments, reaching values around 0.37 and 0.39, respectively, indicating an enhanced antioxidant response under NaCl stress. The CD treatment also showed relatively higher antioxidant activity (0.26–0.30), while SOA exhibited the lowest (0.06–0.10). The combination treatments, such as CD + SOA and *NaCl* + SOA, showed moderate antioxidant levels (0.13–0.17), suggesting a partial mitigation of oxidative stress. In contrast, H_2_O_2_ accumulation was highest in CD + *NaCl* (54.25–58.32), followed by *NaCl* (44.67–46.36) and CD (48.2–51.21), indicating increased oxidative stress. The SOA treatment resulted in the lowest H_2_O_2_ levels (22.63–24.26), suggesting its potential role in reducing oxidative damage. The combined treatments, such as *NaCl* + SOA and CD + SOA, displayed intermediate H_2_O_2_ levels (34.25–37.51), highlighting their role in stress regulation (Fig 11).

### 3.12 Elemental analysis of Cd under different treatments

The Cd concentration varied across treatments, with the control group showing a mean Cd level of 9.979 mg/L, a standard deviation of 0.063, and a relative standard deviation (RSD) of 0.63%. The SOA treatment exhibited a higher mean Cd concentration of 12.65 mg/L, with a standard deviation of 0.521 and an RSD of 4.12%. The CD treatment also elevated the Cd concentration to 13.651 mg/L with a smaller standard deviation (0.125) and RSD of 0.92%. The combined CD + SOA treatment also resulted in a Cd concentration of 10.951 mg/L with a standard deviation of 0.172 and an RSD of 1.57% (Table 1). These results indicate that Cd accumulation was highest under CD treatment, whereas the CD + SOA treatment showed a lower Cd concentration than CD alone. However, SOA alone showed a higher Cd concentration than the control, suggesting that the Cd-modulating effect of SOA was more evident under CD exposure rather than under non-stressed conditions.

**Table 1:** Cadmium (Cd) concentration under different treatments.

Sample ID	Analyte	Mean (mg/L)	Std Dev	% RSD
Control	Cd 228.8	9.979	0.063	0.63
SOA	Cd 228.8	12.65	0.521	4.12
CD	Cd 228.8	13.65	0.125	0.92
CD + SOA	Cd 228.8	10.95	0.172	1.57

## 4. Discussion

*Aspergillus fumigatus* (SOA) application strongly promoted plant growth and stress resistance by alleviating NaCl- and CD-induced stress in okra plants. SOA-treated plants showed an extraordinary 28.9% greater height over the control, while biomass, chlorophyll contents, and the balance of phytohormones were also enriched under stressed conditions. These findings are consistent with earlier research showing that endophytic fungi promote plant adaptation through the control of osmotic stress, ion toxicity, and oxidative damage [51,52]. Interestingly, SOA mitigated the inhibitory effects of NaCl and CD stress on shoot and root growth, consistent with previous reports demonstrating that *Aspergillus fumigatus* enhances plant growth, root development, and biomass production under NaCl stress [27]. Furthermore, SOA-treated plants showed reduced H_2_O_2_ accumulation and higher antioxidant activity, indicating a key function in ROS detoxification and stress acclimation, consistent with reports, when endophytic fungi were inoculated to chickpea and soybean plants under stress conditions [53,54]. Together, these results support SOA’s potential as a stress-tolerance-conferring growth promoter in okra plants.

The endophytic isolate SOA, from *Ziziphus lotus* roots, produced brownish colonies on PDA and reached a maximum biomass yield of 8.76 g/flask in Czapek broth, with its morphology confirmed microscopically. These results are consistent with a previous report showing that endophytic fungi such as *Chaetomium coarctatum* and *Alternaria chlamydospora* showed vigorous growth on PDA media and increased wheat seedling emergence and root elongation under NaCl, suggesting their potential to promote plant growth under stress [55].

SOA showed enhanced hormone production under stress, under CD stress. Similar findings have been reported previously, showing that endophytes exhibit increased hormonal levels under stressful conditions. For example, it has been reported that the endophytes *Penicillium* species and *P. glomerata* regulate host plant hormone levels, lowering ABA content under NaCl and drought stress; in contrast, our own observations indicate relatively stable ABA content during CD stress [10]. Furthermore, it was found that the endophytic fungus *Yarrowia lipolytica* FH1 grew indole-3-acetic acid (IAA) and stimulated maize seedling growth in NaCl stress conditions, which is similar to our findings, where the levels of IAA were increased under CD stress [56]. An increase in the phytohormone levels, such as GA and IAA, was observed in response to CD and NaCl stress in the fungal endophytes, consistent with previous studies on endophytic fungi-mediated stress tolerance [57,58]. Similarly, enhanced antioxidant activity was observed in fungal cultures under stress conditions, leading to increased CAT activity and peroxide production. These findings are consistent with previous studies that endophytes activate plant defense responses against oxidative stress [59,60]. Under CD stress. SOA endophyte culture resulted in high production of primary and secondary metabolites with higher concentrations of sugars, lipids, phenols, flavonoids, proteins, and proline. These findings are consistent with earlier research demonstrating that endophytic fungi augment host plant metabolite accumulation to counteract the effects of stress [61–63]. Comparable increases in phenolic and flavonoid content under stress have also been documented, illustrating their involvement in antioxidative defense mechanisms [64,65]. The observed metabolites indicate that SOA affects stress tolerance, confirming observations of endophyte-mediated stress tolerance in plants [66].

Our results showed that SOA treatment significantly improved okra growth under both normal and stress conditions induced by NaCl and CD. These results align with earlier research indicating that endophytic fungi enhance plant growth during abiotic stress by regulating phytohormone levels and improving nutrient uptake [67,68]. Such growth-enhancing effects of fungal endophytes against NaCl and CD stress have also been seen in other plants, further confirming the significance of endophytes in stress resistance [60,69]. SOA treatment prominently increased chlorophyll and carotenoid content under stress conditions, including chlorophyll, chlorophyll B, and carotenoids, indicating SOA’s capacity to facilitate enhanced photosynthesis in stressed plants. Similar findings have previously been reported; endophytic fungi induce chlorophyll retention under abiotic stress and stimulate carotenoid biosynthesis by increasing antioxidant activity and altering hormonal equilibrium [70,71]. The function of fungal endophytes in chlorophyll stability under stress from NaCl and CD has also been documented in wheat and maize [72]. The SOA treatments notably enhanced shoot and root biomass in stress conditions. Similarly, a notable increase in plant fresh and dry weight was observed in SOA-treated plants under stress conditions. Comparable observations have been reported that endophytic fungi maximize plant biomass under abiotic stress by increasing nutrient intake, regulating antioxidant activity, and modulating phytohormone production [73,74]. The same result has also been reported in maize and wheat by other researchers [30,52], who found that endophytes enhanced shoot and root growth under salinity and CD stress.

Phytohormone analysis indicated large variations among treatments, with SOA’s function to modulate levels of stress-responsive hormones. IAA was greatest in CD + SOA and least in CD + NaCl, indicating SOA’s function in auxin-dependent stress tolerance. GA levels reached a maximum in SOA-treated plants, indicating increased regulation of growth. SA was highest in the control but was reduced in NaCl + SOA, suggesting stress adaptation. ABA, a vital stress-responsive hormone, was highest in the control but was considerably lower in CD + SOA, suggesting SOA’s potential to counteract stress by downregulating ABA levels. These findings agree with earlier research indicating that endophytic fungi regulate phytohormone balance to enhance plant resistance against abiotic stress [7,75]. It has been reported in wheat and maize, where endophyte-mediated hormone regulation enhanced plant tolerance to CD and salinity [6].

Biochemical profiling of secondary metabolites and macromolecules revealed noteworthy treatment-specific differences, highlighting SOA’s contribution to stress adaptation. Flavonoid levels were highest in SOA-treated plants, consistent with earlier observations that endophytic fungi stimulate flavonoid biosynthesis to combat oxidative stress [62,69]. Total soluble sugars were highest in SOA, indicating enhanced osmotic balance during stress, a phenomenon also reported in fungal-assisted stress tolerance in plants [66]. Phenol content followed the same pattern, peaking in SOA, affirming reports of fungal-mediated phenolic compound accumulation for ROS scavenging. Lipid content was highest in NaCl + SOA, consistent with prior research that associated endophyte-mediated stress responses with lateration in lipid metabolism [71]. In contrast, CD + NaCl treatment consistently showed low levels of these biomolecules, reflecting the extreme stress, which was moderated by SOA treatment.

The antioxidant activity and oxidative stress response varied significantly across treatments, aligning with previous studies on fungal-mediated stress tolerance. Elevated antioxidant levels in the NaCl and CD + NaCl treatments indicate a strong oxidative stress response, consistent with a previous study [76], which reported increased antioxidant activity in plants exposed to combined abiotic stresses. Cd-treated plants also exhibited notable antioxidant activity, consistent with previous reports suggesting Cd induces ROS accumulation and antioxidant activation [77]. Interestingly, SOA-treated plants had the lowest antioxidant levels, which agrees with earlier results reported by [78], in which endophytic fungi reduced stress-induced antioxidant overexpression by mitigating oxidative damage. Moderate antioxidant levels in CD + SOA and NaCl + SOA further suggest partial alleviation of oxidative stress, the role of fungal endophytes in balancing ROS levels [61]. H_2_O_2_ accumulation followed a similar trend, with the highest levels in CD + NaCl and NaCl, which agrees with a previous study reporting increased oxidative stress markers [79]. In contrast, SOA-treated plants exhibited the lowest H_2_O_2_ levels, confirming the endophyte’s role in reducing oxidative damage and maintaining cellular homeostasis.

The concentration of Cd differed notably among treatments, suggesting that SOA influenced Cd accumulation in a treatment-dependent manner. The highest Cd concentration was observed under CD treatment, whereas CD + SOA showed a lower Cd concentration than CD alone, indicating that SOA may reduce Cd accumulation or improve Cd detoxification under heavy-metal exposure. However, SOA alone showed a higher Cd concentration than the control; therefore, the Cd-reducing effect should not be interpreted as universal across all conditions. Instead, these results suggest that SOA may modulate Cd uptake, sequestration, or stress-related metal handling, particularly under CD stress. Similar fungal-assisted metal immobilization, biosorption, or sequestration mechanisms have been reported in previous studies on beneficial fungi under metal-stress conditions [80,81].

In this study, *Aspergillus fumigatus* SOA was identified and characterized for its plant growth-promoting potential and its ability to mitigate NaCl- and heavy-metal-induced stress in okra under controlled conditions. The production of growth-related metabolites, including IAA and GA, supports its role in improving plant performance under stress. However, because *A. fumigatus* includes opportunistic strains, strain-level pathogenicity, mycotoxin, and environmental safety assessments are required before considering SOA for agricultural application. Therefore, future work should focus on strain-level biosafety validation, metabolite purification, toxicity assessment, and field-based evaluation before practical deployment.

### Conclusion

The current research shows the potential of SOA to enhance okra growth and better cope with salinity and Cd stress. Treatment with SOA reduces stress-induced growth suppression by increasing photosynthetic pigments and biomass by maintaining phytohormonal balance. The reduced oxidative stress marker and enhanced accumulation of secondary metabolites further support the role of SOA in elevating plant adaptation to unfavorable conditions. SOA treatment reduced Cd accumulation in okra plants compared to those exposed to Cd stress alone, suggesting its potential to alleviate heavy-metal toxicity under stress conditions. While the present study demonstrates the potential of *Aspergillus fumigatus* strain SOA as a plant growth-promoting and stress-modulating agent under controlled conditions, its known biosafety concerns require cautious interpretation. Because *A. fumigatus* includes opportunistic strains, strain-level pathogenicity, mycotoxin, and environmental safety assessments are required before considering SOA for agricultural application.
