# Supplementary material for: Endophytic Aspergillus fumigatus modulates growth, oxidative stress, and cadmium accumulation in okra under salinity and heavy-metal stress
Source: PLoS One. 2026 Aug 13;21(8):e0356384. doi: 10.1371/journal.pone.0356384 (PMC13472373; doi:10.1371/journal.pone.0356384)
Supplement: Supplementary file 1 [file pone.0356384.s002.docx]

Response to Editors’ and Reviewers’ Comments:

PONE-D-26-29102

Endophytic Aspergillus fumigatus Modulates Growth, Oxidative Stress, and Cadmium Accumulation in Okra Under Salinity and Heavy-Metal Stress

PLOS One

Journal Requirements:

1.Please ensure that your manuscript meets PLOS ONE's style requirements, including those for file naming. The PLOS ONE style templates can be found at

https://journals.plos.org/plosone/s/file?id=wjVg/PLOSOne_formatting_sample_main_body.pdf and

https://journals.plos.org/plosone/s/file?id=ba62/PLOSOne_formatting_sample_title_authors_affiliations.pdf

Response: We have revised the manuscript and file names in accordance with the PLOS ONE style requirements.

2. Thank you for stating the following financial disclosure:

“The authors extend their appreciation to the ongoing Research Funding Program (ORF-2026-1492), King Saud University, Riyadh, Saudi Arabia.”

Please state what role the funders took in the study. If the funders had no role, please state: "The funders had no role in study design, data collection and analysis, decision to publish, or preparation of the manuscript."

If this statement is not correct you must amend it as needed.

Please include this amended Role of Funder statement in your cover letter; we will change the online submission form on your behalf.

Response: We have included the amended Role of Funder statement in the revised cover letter, as requested.

3. Thank you for stating the following financial disclosure:

““The authors extend their appreciation to the ongoing Research Funding Program (ORF-2026-1492), King Saud University, Riyadh, Saudi Arabia.”

We note that one or more of the authors is affiliated with the funding organization, indicating the funder may have had some role in the design, data collection, analysis or preparation of your manuscript for publication; in other words, the funder played an indirect role through the participation of the co-authors. If the funding organization did not play a role in the study design, data collection and analysis, decision to publish, or preparation of the manuscript and only provided financial support in the form of authors' salaries and/or research materials, please do the following:

Review your statements relating to the author contributions, and ensure you have specifically and accurately indicated the role(s) that these authors had in your study. These amendments should be made in the online form.

Confirm in your cover letter that you agree with the following statement, and we will change the online submission form on your behalf:

“The funder provided support in the form of salaries for authors [insert relevant initials], but did not have any additional role in the study design, data collection and analysis, decision to publish, or preparation of the manuscript. The specific roles of these authors are articulated in the ‘author contributions’ section.

Response: We have carefully reviewed and accurately updated the author contributions in the online submission form. We also confirm our agreement with the requested Role of Funder statement and have included it in the revised cover letter with the relevant author initials. In addition, PLOS’s inclusivity in the global research questionnaire has been filled out and uploaded via the online editorial manager.

4. Please include a copy of Table 1 which you refer to in your text on page 13.

Response: Table 1 has now been included in the manuscript under Section 3.12.

5. If the reviewer comments include a recommendation to cite specific previously published works, please review and evaluate these publications to determine whether they are relevant and should be cited. There is no requirement to cite these works unless the editor has indicated otherwise.

Response: We have evaluated the recommended publications and cited only those that are directly relevant to the present study.

Additional Editor Comments:

Dear Authors,

The reviewers consider the topic relevant and potentially valuable. However, substantial revisions are required before the manuscript can be reconsidered.

Please carefully address the inconsistencies in experimental replication, clearly specifying the number of pots, plants per pot, biological replicates, and technical replicates. All reported units and calculations, particularly those for gibberellic acid, other phytohormones, and cadmium concentrations, must be verified. The surface-sterilization reagent should also be clarified, as sodium hypochlorite rather than sodium chloride is normally used.

**Response:** We have thoroughly revised the Materials and Methods section to improve clarity, consistency, and accuracy.

First, the experimental design has been clarified by explicitly specifying the number of pots used per treatment, the number of plants maintained in each pot, and the number of biological replicates included in each experiment. These revisions ensure that the experimental replication strategy is transparent and reproducible.

Second, all reported units, calculations, and data presentations have been carefully rechecked throughout the manuscript. In particular, the units and calculations for gibberellic acid, other phytohormones, and cadmium concentrations have been verified and corrected where necessary to ensure consistency and accuracy across the text, tables, and figures.

Finally, we appreciate the reviewer for identifying the error regarding the surface-sterilization reagent. This was a typographical mistake in the manuscript. The text has been corrected to indicate the use of sodium hypochlorite (NaOCl), which was the reagent actually used for surface sterilization, rather than sodium chloride (NaCl).

The conclusions regarding cadmium uptake should be revised to reflect that the fungal treatment reduced cadmium accumulation under heavy-metal stress relative to the heavy-metal treatment alone, rather than implying a universal reduction in cadmium uptake. Please also strengthen the statistical reporting by providing replicate numbers, measures of variability, significance indicators, and relevant statistical details in all figures and tables.
Response: Thanks for this valuable suggestion. The conclusion portion regarding cadmium was revised and highlighted. Other relevant statistical details were also added where necessary.

In addition, the manuscript requires thorough language editing, standardization of abbreviations, treatment labels, units, decimal precision, and figure legends. The Introduction and Discussion should be shortened to remove repetition, clearly state the novelty of the study, and provide greater mechanistic interpretation of phytohormone regulation, antioxidant responses, and possible cadmium immobilization or sequestration. Finally, all references should be carefully audited for relevance, accuracy, publication status, and correct citation placement.

Response: In response to these comments, we have carefully revised the manuscript throughout.

Specifically, the manuscript has undergone thorough language editing to improve grammar, readability, and scientific clarity. Abbreviations, treatment labels, units, decimal precision, and figure legends have been standardized throughout the manuscript to ensure consistency and compliance with the journal's formatting requirements.

The Introduction and Discussion sections have been substantially revised and condensed to eliminate repetitive information. The novelty of the study has been stated more explicitly in the Introduction section.

In the Discussion, we have strengthened the mechanistic interpretation of the observed responses by providing additional explanation of the roles of phytohormonal regulation, antioxidant defense systems, and secondary metabolite accumulation in stress tolerance.

Finally, all references have been carefully reviewed and updated to ensure their relevance, accuracy, publication status, and appropriate citation placement throughout the manuscript.

**Reviewer #1:**

This manuscript reports on the isolation of the endophytic fungus Aspergillus fumigatus (strain SOA) from Ziziphus lotus roots and its ability to enhance growth, modulate phytohormone profiles, elevate antioxidant defenses, and reduce cadmium accumulation in okra (Abelmoschus esculentus) under salt and heavy-metal stress conditions. The study addresses a relevant agricultural challenge, employs a reasonably comprehensive experimental design, and presents generally well-supported conclusions. The manuscript is suitable for publication in PLOS ONE following minor revisions as detailed below.

Minor comments:

1. Line 64: “soil salinization is increasing by nearly 10% per year” — this statement requires a citation. Please add the appropriate reference.

Response: Thank you for your valuable suggestion. We have added an appropriate reference to support the statement "soil salinization is increasing by nearly 10% per year" in Line 64 of the revised manuscript.

2. Lines 279–286 (Section 3.4): Metabolite values are reported with varying decimal precision (e.g., 17.64, 0.0769). Please standardize significant figures consistently throughout the Results.

Response: Thank you for your valuable comment. We have carefully reviewed the Results section and standardized the numerical values by using consistent decimal precision/significant figures throughout the manuscript.

3. Line 492: “alt” appears to be a typographical error for “Salt.” Please correct.

Response: Thank you for your careful observation. The typographical error has been corrected, and "alt" has been changed to "Salt".

4. Figure 6 caption refers to measurements over “three weeks” in the text, while figure panels show “1 week,” “2 weeks,” and “7 weeks.” Reconcile caption and figure labels.

Response: Thank you for pointing out this inconsistency. We have carefully reviewed Figure 6 and corrected the discrepancy between the figure caption and the panel labels. The caption has been revised to accurately reflect the time points presented in the figure (1 week, 2 weeks, and 7 weeks), ensuring consistency between the figure and its description throughout the manuscript.

5. Figure 11 legend (line 758) references “IBH + salt stress” — “IBH” appears to be a typographical error; likely intended as “HM” or “HM+Salt.” Please correct.

Response: Thank you for bringing this typographical error to our attention. We have corrected the Figure 11 legend by replacing the incorrect term “IBH + salt stress” with the correct treatment, HM + Salt.

6. Reference 10 (Warrier et al., 2013) is cited in the context of global temperature increase predictions but appears to be a paper on salicylic acid estimation in Eucalyptus. This appears to be a misplaced citation. Please verify and replace with an appropriate climate science reference.

Response: Thank you for pointing out this error. We carefully reviewed the cited reference and confirmed that it was incorrectly cited in the context of global temperature increase predictions. The misplaced citation has been replaced with an appropriate reference that accurately supports the statement.

7. Reference 73 (Zhang et al., 2026) is listed with a publication year of 2026. If this is an in-press or advance online publication, please provide the DOI and indicate “in press” or “advance online publication” as appropriate per PLOS ONE style.

Response: The publication is already online. The page number and DOI has been added to the reference in the list.

8. The abbreviation “FCF” (fungal culture filtrate) used in Section 2.5 (line 178) is not defined at first use. Please define it upon first appearance in the text.

Response: Thank you for your valuable comment. We have defined the abbreviation fungal culture filtrate (FCF) at its first occurrence in Section 2.5 to improve clarity.

9. Section 2.6 lists eight treatments but the Discussion (line 383) refers to “Aspergillus and Trichoderma species” as a comparative context without a corresponding treatment in this study. This reference is fine but could be slightly misleading; consider adding a brief clarifying phrase that this is a literature comparison.

Response: Thank you for your valuable suggestion. Since Aspergillus fumigatus was one of the fungal species used in the present study, we specifically referred to Aspergillus fumigatus rather than the broader genus Aspergillus and added an appropriate supporting reference.

10. The keywords include “zero hunger,” which, while thematically relevant to food security, is an SDG policy term rather than a scientific keyword. Authors may wish to consider replacing it with a more specific scientific term (e.g., “food security,” “crop productivity,” or “sustainable agriculture”) for better indexing.

Response: Thank you for your valuable suggestion. We have replaced zero hunger with "sustainable agriculture," which more accurately reflects the scientific focus of the study. The keyword list has been revised accordingly.

**Reviewer #2:**

Title: Endophytic Aspergillus fumigatus Modulates Growth, Oxidative Stress, and Cadmium Accumulation in Okra Under Salinity and Heavy-Metal Stress
The manuscript investigates the role of an endophytic fungal strain, Aspergillus fumigatus SOA, in improving okra growth under salinity and cadmium stress. The authors isolated the fungus from Ziziphus lotus roots and evaluated its plant growth-promoting traits, including hormone production, antioxidant response, secondary metabolites, and cadmium modulation. The study reports that SOA improved plant height, biomass, chlorophyll content, and phytohormonal balance under stress conditions. It also suggests that SOA may help reduce oxidative damage and modulate cadmium uptake in okra. Overall, the work addresses a relevant issue in sustainable agriculture and stress physiology.
The topic is timely because salinity and heavy-metal contamination are serious threats to crop productivity. The use of an endophytic fungus as a biological tool for improving okra tolerance is interesting and practically relevant. The manuscript includes a wide range of physiological, biochemical, hormonal, antioxidant, and elemental analyses, which gives the study good experimental coverage. Molecular identification of the fungal isolate through ITS sequencing strengthens the reliability of the work.

1. The abstract is generally clear, but it would benefit from more precise reporting of the experimental conditions. The authors should briefly mention the salt and cadmium stress levels and include one or two key numerical findings beyond plant height.

Response: We sincerely thank the reviewer for this valuable suggestion. The abstract has been revised to provide a clearer description of the experimental conditions by specifying the concentrations of NaCl and Cd used to impose salt and cadmium stress. In addition, we have included key quantitative findings beyond plant height, such as improvements in biomass and chlorophyll content (as appropriate), to better highlight the significance of the study. These revisions provide a more informative and balanced summary of the experimental design and major outcomes.

2. The introduction contains some repetition about abiotic stress, salinity, and physiological damage. The authors should shorten these repeated parts and focus more directly on the specific gap: the limited evidence on endophytic fungi for okra under combined salt and cadmium stress.

Response: Thank you for this valuable suggestion. The Introduction has been carefully revised to improve clarity and avoid repetition. Repeated descriptions of abiotic stress, salinity, and their associated physiological effects have been condensed, resulting in a more concise and focused narrative. Greater emphasis has been placed on the specific research gap, namely the limited evidence regarding the use of endophytic fungi, particularly Aspergillus fumigatus, to enhance okra tolerance under the combined stress of salinity and cadmium.

3. The novelty of the work should be stated more clearly at the end of the introduction. It should be clear whether the novelty lies in the fungal strain, the plant model, the combined stress treatment, or the multi-parameter evaluation.

Response: Thank you for this valuable suggestion. The novelty of the study has been explicitly stated at the end of the Introduction. The revised text clearly highlights that the originality of this work lies in evaluating an endophytic Aspergillus fumigatus strain isolated from Ziziphus lotus for improving okra tolerance under combined salinity and cadmium stress.

4. Some methodological wording needs correction. For example, phrases such as “misleading morphological features,” “chapel medium,” and “duplicate the osmotic pressure” are unclear and should be revised for scientific accuracy.

Response: Thank you for your constructive comment. We have revised this section to improve its scientific clarity and precision.

5. The fungal isolation section is somewhat repetitive. The authors describe PDA sub-culturing and Czapek broth culturing more than once. This section should be streamlined to improve readability.

Response: Thank you for your valuable suggestion. We have carefully revised the fungal isolation section to improve its clarity and readability. Repetitive descriptions of PDA sub-culturing and Czapek broth culturing have been removed.

6. The surface sterilization protocol should be checked. The manuscript mentions 70% ethanol followed by NaCl 2.0%. Usually, sodium hypochlorite is used for surface sterilization, not sodium chloride. If NaCl was actually used, the authors should justify it; otherwise, this should be corrected.

Response: Thank you for your careful observation. The reagent used for surface sterilization was 2% sodium hypochlorite (NaOCl), not 2% sodium chloride (NaCl). This was a typographical mistake in the manuscript. We have corrected the text accordingly to accurately describe the surface sterilization protocol (70% ethanol followed by 2% sodium hypochlorite.

7. Units should be checked throughout the manuscript. For example, GA is reported as mg/mL in fungal profiling, while other hormones are reported in µg/mL, and plant hormones are later reported as mg/g FW. These unit differences should be verified and made consistent.

Response: We have carefully reviewed all units reported throughout the manuscript and corrected any inconsistencies. We confirmed that the units used for fungal metabolite profiling and plant tissue hormone analysis represent different measurement types and have ensured that they are reported appropriately and consistently. Specifically, phytohormone concentrations in fungal culture filtrates are now presented using uniform concentration units (e.g., µg/mL), while endogenous phytohormone levels in plant tissues are consistently expressed on a fresh weight basis (e.g., µg/g FW). In addition, all tables, figures, and the Materials and Methods section were carefully checked to ensure that units are accurate, standardized, and consistent throughout the revised manuscript

8. The discussion is relevant but somewhat repetitive. Several paragraphs restate the results and then compare them with previous studies. The authors should reduce repetition and add a deeper mechanistic explanation of hormone regulation, ROS detoxification, and possible Cd sequestration or immobilization.

Response: We have thoroughly revised the Discussion to improve its scientific depth and readability. Specifically, repetitive descriptions of the experimental results have been removed or substantially condensed, and greater emphasis has been placed on interpreting the underlying physiological and biochemical mechanisms rather than restating the findings.

To strengthen the Discussion, we incorporated a more detailed mechanistic explanation of how endophytic fungal inoculation regulates phytohormone homeostasis under cadmium (Cd) stress. The revised text explains that increased levels of growth-promoting hormones, including indole-3-acetic acid (IAA) and gibberellic acid (GA), together with reduced abscisic acid (ABA) accumulation, contribute to sustained cell division, root development, photosynthetic activity, and stress adaptation under Cd toxicity.

9. The figures and legends require careful proofreading. Treatment labels, units, abbreviations, and spelling should be checked throughout. Any inconsistent terms, such as HM, heavy metal, Cd, salt, NaCl, and SOA, should be standardized.

Response: In response, all figures and figure legends have been thoroughly proofread and revised to improve consistency, accuracy, and clarity. Treatment labels, units of measurement, abbreviations, and spelling have been carefully checked throughout the manuscript.

Specifically, inconsistent terminology has been standardized across all figures, legends, tables, and the main text. The terms "HM" and "heavy metal" have been consistently replaced with "cadmium (Cd)" where appropriate, while "salt" has been revised to the specific treatment "NaCl" only where salinity is discussed and removed where not applicable to avoid confusion. Similarly, the abbreviation "SOA" has been used consistently throughout the manuscript after its first definition, and all other abbreviations have been defined at their first occurrence and applied uniformly thereafter.

**Reviewer #3:**

1. Inconsistency in Experimental Replication
The manuscript reports (Lines 242–243)"All experiments were performed in three biological replicates"
However, earlier:
"All treatments had six plants..." (Lines 177–184) and "Each replicated six times" (Line 183)
These statements are contradictory.
Number of pots per treatment
Number of plants per pot
Number of biological replicates
Number of technical replicates

Response: The text regarding biological and technical replicates were verified. It has been corrected throughout the manuscript to accurately reflect the experimental design. We have also revised the Materials and Methods section to clearly describe the number of pots, plants per pot, biological replicates, and the replication used for statistical analyses, thereby eliminating any ambiguity and ensuring consistency throughout the manuscript.

2. Units Need Verification
Several hormone concentrations appear unusually high:
Examples:
GA = 20–28 mg/mL
IAA = 24–31 µg/mL
For fungal culture filtrates, GA concentrations in mg/mL are extremely high and likely incorrect.
Check original calculations and units carefully.
Possible intended units:
µg/mL
ng/mL
This requires verification before publication.

Response: We have carefully reviewed all units reported throughout the manuscript and corrected any inconsistencies. We confirmed that the units used for fungal metabolite profiling represent different types of measurements and have ensured that they are reported appropriately and consistently. GA measurement units were verified and corrected.

3. Cadmium Uptake Interpretation

In Results:

Control = 9.979 mg/L Cd
SOA = 12.65 mg/L Cd
HM = 13.65 mg/L Cd
HM+SOA = 10.95 mg/L Cd

The conclusion:
"SOA reduced heavy metal toxicity by modulating cadmium uptake"is only partially supported.
SOA alone increased Cd accumulation compared with control.
Recommended wording:
"SOA reduced Cd accumulation under heavy-metal stress conditions compared with HM treatment alone." instead of a broad statement that SOA always reduces Cd uptake.

Response: The conclusion section were modified and re-written, specifically the Cd accumulation statement, as suggested.

4. Surface Sterilization Procedure
Line 120 it is mentioned "treated with 70% ethanol for 1 minute, NaCl (2.0%) for 2 minutes"
Normally fungal isolation protocols use “Sodium hypochlorite (NaOCl)” not NaCl.
It should be mentioned clearly that whether NaCl was actually used, or NaOCl.

Response: Thank you for your careful observation. The reagent used for surface sterilization was 2% sodium hypochlorite (NaOCl), not 2% sodium chloride (NaCl). This was a typographical mistake in the manuscript. We have corrected the text accordingly to accurately describe the surface sterilization protocol (70% ethanol followed by 2% sodium hypochlorite.

5. Major Language Errors
Abstract

Line 42
Current:
"with 28.9% increase in plant height"
Correct:
"with a 28.9% increase in plant height"

Line 49
Current:
"enhance plants' resilience"
Better:
"enhance plant resilience"
Introduction
Line 58
Current:
"Crop productivity was adversely affected"
Should be:
"Crop productivity is adversely affected"
Line 80
Current:
"biomoleculessuch"
Correct:
"biomolecules such"

Line 84
Current:
"up regulated"
Correct:
"upregulated"
Methodology
Line 133
Current:
"The misleading morphological features"
Incorrect wording.
Suggested:
"The morphological characteristics of the isolates"

Line 138
Current:
"To duplicate the osmotic pressure of the fungal cells within the chapel medium"
Problems:
duplicate → simulate
chapel medium → Czapek medium
Suggested:
"To simulate osmotic stress in Czapek medium..."

Line 145
Current:
"To promote the host plant growth"
Better:
"To promote host plant growth"

Line 166
Current:
"Abelmoschus esculentus (L.) genotype seeds"
Incorrect.
Suggested:
"Seeds of Abelmoschus esculentus (L.)"
or
"Seeds of an Abelmoschus esculentus genotype"

Line 203
Current:
"The method determined Gibberellin acid and ABA"
Correct:
"Gibberellic acid and ABA were quantified according to..."

Response: Thank you for your careful review and valuable suggestions regarding the language and grammar. We have thoroughly revised the manuscript to address all of the points raised.

6. Scientific Writing Issues
There are many repetitive sentences in Discussion.
The Discussion repeatedly restates numerical results instead of interpreting them.
Examples:
Lines 397–401
Lines 410–417
Lines 425–429
Lines 444–454
The discussion should focus more on:
Mechanisms, comparison with previous studies, novelty of findings, and less on repeating Results.
Overuse of "The same results have been reported"
Repeated phrases:
"The same results have been documented..."
"The same result has also been reported..."
"The same trends have been reported..."
Use alternatives:
"Similarly,"
"Consistent with previous reports,"
"These findings agree with..."
"Comparable observations have been reported..."

Response: Thank you for this valuable comment. We have thoroughly revised the Discussion section to improve its scientific interpretation and reduce repetition. Repetitive descriptions of numerical results have been removed.

7. Statistical Reporting Issues
The manuscript reports many numerical differences but does not consistently indicate:
p-values
standard errors
significance letters
confidence intervals
Ensure that all figures and tables include:
Mean ± SE (or SD)
Number of replicates (n)
Statistical grouping letters

Response: We have carefully revised the statistical reporting throughout the manuscript to improve clarity and transparency. Specifically, all figures and tables have been updated to present the data as mean ± standard error (SE) (or standard deviation (SD), where appropriate), and the number of biological replicates (n) has been clearly indicated in the figure legends and table footnotes.

8. Reference Issues
Some references appear unrelated or weakly connected to the cited statements.
Examples:
Ref. 10 (salicylic acid estimation) is cited in climate-change discussion.
Verify all citations carefully because there may be citation-management errors.
A full reference audit is recommended before resubmission.

Response: Thank you for bringing this to our attention. We carefully reviewed all references cited throughout the manuscript to ensure their relevance and accuracy. The inappropriate citation to Ref. 10 in the climate change discussion has been corrected by replacing it with a more relevant reference that directly supports the statement in question.

Sincerely

Prof. Dr. Ayaz Ahmad

On behlf of all authors
