# Supplementary material for: Endophytic Aspergillus fumigatus modulates growth, oxidative stress, and cadmium accumulation in okra under salinity and heavy-metal stress
Source: PLoS One. 2026 Aug 13;21(8):e0356384. doi: 10.1371/journal.pone.0356384 (PMC13472373; doi:10.1371/journal.pone.0356384)
Supplement: Peer review history

# Peer review: Decision Letter 0

**DOI:** 10.1371/journal.pone.0356384.r001

**Authors:** Wajid Zaman

3 Jul 2026

Dear Dr. Ahmad,

Thank you for submitting your manuscript to PLOS ONE. After careful consideration, we feel that it has merit but does not fully meet PLOS ONE’s publication criteria as it currently stands. Therefore, we invite you to submit a revised version of the manuscript that addresses the points raised during the review process.Please submit your revised manuscript by Sep 01 2026 11:59PM. If you will need more time than this to complete your revisions, please reply to this message or contact the journal office at plosone@plos.org. When you’re ready to submit your revision, log on to https://www.editorialmanager.com/pone/ and select the ‘Submissions Needing Revision’ folder to locate your manuscript file.

- A letter that responds to each point raised by the academic editor and reviewer(s). You should upload this letter as a separate file labeled ‘Response to Reviewers’.
- A marked-up copy of your manuscript that highlights changes made to the original version. You should upload this as a separate file labeled ‘Revised Manuscript with Track Changes’.
- An unmarked version of your revised paper without tracked changes. You should upload this as a separate file labeled ‘Manuscript’.

If you would like to make changes to your financial disclosure, please include your updated statement in your cover letter. Guidelines for resubmitting your figure files are available below the reviewer comments at the end of this letter.

If applicable, we recommend that you deposit your laboratory protocols in protocols.io to enhance the reproducibility of your results. Protocols.io assigns your protocol its own identifier (DOI) so that it can be cited independently in the future. For instructions see: https://journals.plos.org/plosone/s/submission-guidelines#loc-laboratory-protocols. Additionally, PLOS ONE offers an option for publishing peer-reviewed Lab Protocol articles, which describe protocols hosted on protocols.io. Read more information on sharing protocols at https://plos.org/protocols?utm_medium=editorial-email&utm_source=authorletters&utm_campaign=protocols.

As the corresponding author, your ORCID iD is verified in the submission system and will appear in the published article. PLOS supports the use of ORCID, and we encourage all coauthors to register for an ORCID iD and use it as well. Please encourage your coauthors to verify their ORCID iD within the submission system before final acceptance, as unverified ORCID iDs will not appear in the published article. *Only*  the individual author can complete the verification step; PLOS staff *cannot*  verify ORCID iDs on behalf of authors.

We look forward to receiving your revised manuscript.

Kind regards,

Wajid Zaman

Academic Editor

PLOS One

Journal Requirements:

When submitting your revision, we need you to address these additional requirements.

1.Please ensure that your manuscript meets PLOS ONE’s style requirements, including those for file naming. The PLOS ONE style templates can be found at

https://journals.plos.org/plosone/s/file?id=wjVg/PLOSOne_formatting_sample_main_body.pdf and

https://journals.plos.org/plosone/s/file?id=ba62/PLOSOne_formatting_sample_title_authors_affiliations.pdf

2. Thank you for stating the following financial disclosure:

“The authors extend their appreciation to the ongoing Research Funding Program (ORF-2026-1492), King Saud University, Riyadh, Saudi Arabia.”

Please state what role the funders took in the study.  If the funders had no role, please state: "The funders had no role in study design, data collection and analysis, decision to publish, or preparation of the manuscript."

If this statement is not correct you must amend it as needed.

Please include this amended Role of Funder statement in your cover letter; we will change the online submission form on your behalf.

3. Thank you for stating the following financial disclosure:

““The authors extend their appreciation to the ongoing Research Funding Program (ORF-2026-1492), King Saud University, Riyadh, Saudi Arabia.”

We note that one or more of the authors is affiliated with the funding organization, indicating the funder may have had some role in the design, data collection, analysis or preparation of your manuscript for publication; in other words, the funder played an indirect role through the participation of the co-authors. If the funding organization did not play a role in the study design, data collection and analysis, decision to publish, or preparation of the manuscript and only provided financial support in the form of authors’ salaries and/or research materials, please do the following:

1. Review your statements relating to the author contributions, and ensure you have specifically and accurately indicated the role(s) that these authors had in your study. These amendments should be made in the online form.

2. Confirm in your cover letter that you agree with the following statement, and we will change the online submission form on your behalf:

“The funder provided support in the form of salaries for authors [insert relevant initials], but did not have any additional role in the study design, data collection and analysis, decision to publish, or preparation of the manuscript. The specific roles of these authors are articulated in the ‘author contributions’ section.

4. Please include a copy of Table 1 which you refer to in your text on page 13.

5. If the reviewer comments include a recommendation to cite specific previously published works, please review and evaluate these publications to determine whether they are relevant and should be cited. There is no requirement to cite these works unless the editor has indicated otherwise.

Additional Editor Comments:

Dear Authors,

The reviewers consider the topic relevant and potentially valuable. However, substantial revisions are required before the manuscript can be reconsidered.

Please carefully address the inconsistencies in experimental replication, clearly specifying the number of pots, plants per pot, biological replicates, and technical replicates. All reported units and calculations, particularly those for gibberellic acid, other phytohormones, and cadmium concentrations, must be verified. The surface-sterilization reagent should also be clarified, as sodium hypochlorite rather than sodium chloride is normally used.

The conclusions regarding cadmium uptake should be revised to reflect that the fungal treatment reduced cadmium accumulation under heavy-metal stress relative to the heavy-metal treatment alone, rather than implying a universal reduction in cadmium uptake. Please also strengthen the statistical reporting by providing replicate numbers, measures of variability, significance indicators, and relevant statistical details in all figures and tables.

In addition, the manuscript requires thorough language editing, standardization of abbreviations, treatment labels, units, decimal precision, and figure legends. The Introduction and Discussion should be shortened to remove repetition, clearly state the novelty of the study, and provide greater mechanistic interpretation of phytohormone regulation, antioxidant responses, and possible cadmium immobilization or sequestration. Finally, all references should be carefully audited for relevance, accuracy, publication status, and correct citation placement.

A detailed point-by-point response to all reviewer comments and a clearly marked revised manuscript should be provided with the resubmission.

[Note: HTML markup is below. Please do not edit.]

Reviewers’ comments:

Reviewer’s Responses to Questions

**Comments to the Author**

1. Is the manuscript technically sound, and do the data support the conclusions?

Reviewer #1: Yes

Reviewer #2: Yes

Reviewer #3: Yes

**********

2. Has the statistical analysis been performed appropriately and rigorously?

Reviewer #1: Yes

Reviewer #2: Yes

Reviewer #3: Yes

**********

3. Have the authors made all data underlying the findings in their manuscript fully available?

The PLOS Data policy

Reviewer #1: Yes

Reviewer #2: Yes

Reviewer #3: Yes

**********

4. Is the manuscript presented in an intelligible fashion and written in standard English?

Reviewer #1: Yes

Reviewer #2: Yes

Reviewer #3: Yes

**********

Reviewer #1: This manuscript reports on the isolation of the endophytic fungus Aspergillus fumigatus (strain SOA) from Ziziphus lotus roots and its ability to enhance growth, modulate phytohormone profiles, elevate antioxidant defenses, and reduce cadmium accumulation in okra (Abelmoschus esculentus) under salt and heavy-metal stress conditions. The study addresses a relevant agricultural challenge, employs a reasonably comprehensive experimental design, and presents generally well-supported conclusions. The manuscript is suitable for publication in PLOS ONE following minor revisions as detailed below.

Minor comments:

1. Line 64: “soil salinization is increasing by nearly 10% per year” — this statement requires a citation. Please add the appropriate reference.

2. Lines 279–286 (Section 3.4): Metabolite values are reported with varying decimal precision (e.g., 17.64, 0.0769). Please standardize significant figures consistently throughout the Results.

3. Line 492: “alt” appears to be a typographical error for “Salt.” Please correct.

4. Figure 6 caption refers to measurements over “three weeks” in the text, while figure panels show “1 week,” “2 weeks,” and “7 weeks.” Reconcile caption and figure labels.

5. Figure 11 legend (line 758) references “IBH + salt stress” — “IBH” appears to be a typographical error; likely intended as “HM” or “HM+Salt.” Please correct.

6. Reference 10 (Warrier et al., 2013) is cited in the context of global temperature increase predictions but appears to be a paper on salicylic acid estimation in Eucalyptus. This appears to be a misplaced citation. Please verify and replace with an appropriate climate science reference.

7. Reference 73 (Zhang et al., 2026) is listed with a publication year of 2026. If this is an in-press or advance online publication, please provide the DOI and indicate “in press” or “advance online publication” as appropriate per PLOS ONE style.

8. The abbreviation “FCF” (fungal culture filtrate) used in Section 2.5 (line 178) is not defined at first use. Please define it upon first appearance in the text.

9. Section 2.6 lists eight treatments but the Discussion (line 383) refers to “Aspergillus and Trichoderma species” as a comparative context without a corresponding treatment in this study. This reference is fine but could be slightly misleading; consider adding a brief clarifying phrase that this is a literature comparison.

10. The keywords include “zero hunger,” which, while thematically relevant to food security, is an SDG policy term rather than a scientific keyword. Authors may wish to consider replacing it with a more specific scientific term (e.g., “food security,” “crop productivity,” or “sustainable agriculture”) for better indexing.

Reviewer #2: Title: Endophytic Aspergillus fumigatus Modulates Growth, Oxidative Stress, and Cadmium Accumulation in Okra Under Salinity and Heavy-Metal Stress

The manuscript investigates the role of an endophytic fungal strain, Aspergillus fumigatus SOA, in improving okra growth under salinity and cadmium stress. The authors isolated the fungus from Ziziphus lotus roots and evaluated its plant growth-promoting traits, including hormone production, antioxidant response, secondary metabolites, and cadmium modulation. The study reports that SOA improved plant height, biomass, chlorophyll content, and phytohormonal balance under stress conditions. It also suggests that SOA may help reduce oxidative damage and modulate cadmium uptake in okra. Overall, the work addresses a relevant issue in sustainable agriculture and stress physiology.

The topic is timely because salinity and heavy-metal contamination are serious threats to crop productivity. The use of an endophytic fungus as a biological tool for improving okra tolerance is interesting and practically relevant. The manuscript includes a wide range of physiological, biochemical, hormonal, antioxidant, and elemental analyses, which gives the study good experimental coverage. Molecular identification of the fungal isolate through ITS sequencing strengthens the reliability of the work.

1. The abstract is generally clear, but it would benefit from more precise reporting of the experimental conditions. The authors should briefly mention the salt and cadmium stress levels and include one or two key numerical findings beyond plant height.

2. The introduction contains some repetition about abiotic stress, salinity, and physiological damage. The authors should shorten these repeated parts and focus more directly on the specific gap: the limited evidence on endophytic fungi for okra under combined salt and cadmium stress.

3. The novelty of the work should be stated more clearly at the end of the introduction. It should be clear whether the novelty lies in the fungal strain, the plant model, the combined stress treatment, or the multi-parameter evaluation.

4. Some methodological wording needs correction. For example, phrases such as “misleading morphological features,” “chapel medium,” and “duplicate the osmotic pressure” are unclear and should be revised for scientific accuracy.

5. The fungal isolation section is somewhat repetitive. The authors describe PDA sub-culturing and Czapek broth culturing more than once. This section should be streamlined to improve readability.

6. The surface sterilization protocol should be checked. The manuscript mentions 70% ethanol followed by NaCl 2.0%. Usually, sodium hypochlorite is used for surface sterilization, not sodium chloride. If NaCl was actually used, the authors should justify it; otherwise, this should be corrected.

7. Units should be checked throughout the manuscript. For example, GA is reported as mg/mL in fungal profiling, while other hormones are reported in µg/mL, and plant hormones are later reported as mg/g FW. These unit differences should be verified and made consistent.

8. The discussion is relevant but somewhat repetitive. Several paragraphs restate the results and then compare them with previous studies. The authors should reduce repetition and add a deeper mechanistic explanation of hormone regulation, ROS detoxification, and possible Cd sequestration or immobilization.

9. The figures and legends require careful proofreading. Treatment labels, units, abbreviations, and spelling should be checked throughout. Any inconsistent terms, such as HM, heavy metal, Cd, salt, NaCl, and SOA, should be standardized.

Reviewer #3: 1. Inconsistency in Experimental Replication

The manuscript reports (Lines 242–243)"All experiments were performed in three biological replicates"

However, earlier:

"All treatments had six plants..." (Lines 177–184) and "Each replicated six times" (Line 183)

These statements are contradictory.

Number of pots per treatment

Number of plants per pot

Number of biological replicates

Number of technical replicates

2. Units Need Verification

Several hormone concentrations appear unusually high:

Examples:

GA = 20–28 mg/mL

IAA = 24–31 µg/mL

For fungal culture filtrates, GA concentrations in mg/mL are extremely high and likely incorrect.

Check original calculations and units carefully.

Possible intended units:

µg/mL

ng/mL

This requires verification before publication.

3. Cadmium Uptake Interpretation

In Results:

Control = 9.979 mg/L Cd

SOA = 12.65 mg/L Cd

HM = 13.65 mg/L Cd

HM+SOA = 10.95 mg/L Cd

The conclusion:

"SOA reduced heavy metal toxicity by modulating cadmium uptake"is only partially supported.

SOA alone increased Cd accumulation compared with control.

Recommended wording:

"SOA reduced Cd accumulation under heavy-metal stress conditions compared with HM treatment alone." instead of a broad statement that SOA always reduces Cd uptake.

4. Surface Sterilization Procedure

Line 120 it is mentioned "treated with 70% ethanol for 1 minute, NaCl (2.0%) for 2 minutes"

Normally fungal isolation protocols use “Sodium hypochlorite (NaOCl)” not NaCl.

It should be mentioned clearly that whether NaCl was actually used, or NaOCl.

5. Major Language Errors

Abstract

Line 42

Current:

“with 28.9% increase in plant height”

Correct:

“with a 28.9% increase in plant height”

Line 49

Current:

“enhance plants’ resilience”

Better:

"enhance plant resilience"

Introduction

Line 58

Current:

"Crop productivity was adversely affected"

Should be:

"Crop productivity is adversely affected"

Line 80

Current:

"biomoleculessuch"

Correct:

"biomolecules such"

Line 84

Current:

"up regulated"

Correct:

"upregulated"

Methodology

Line 133

Current:

"The misleading morphological features"

Incorrect wording.

Suggested:

"The morphological characteristics of the isolates"

Line 138

Current:

"To duplicate the osmotic pressure of the fungal cells within the chapel medium"

Problems:

duplicate → simulate

chapel medium → Czapek medium

Suggested:

"To simulate osmotic stress in Czapek medium..."

Line 145

Current:

"To promote the host plant growth"

Better:

"To promote host plant growth"

Line 166

Current:

"Abelmoschus esculentus (L.) genotype seeds"

Incorrect.

Suggested:

"Seeds of Abelmoschus esculentus (L.)"

or

"Seeds of an Abelmoschus esculentus genotype"

Line 203

Current:

"The method determined Gibberellin acid and ABA"

Correct:

"Gibberellic acid and ABA were quantified according to..."

6. Scientific Writing Issues

There are many repetitive sentences in Discussion.

The Discussion repeatedly restates numerical results instead of interpreting them.

Examples:

Lines 397–401

Lines 410–417

Lines 425–429

Lines 444–454

The discussion should focus more on:

Mechanisms, comparison with previous studies, novelty of findings, and less on repeating Results.

Overuse of "The same results have been reported"

Repeated phrases:

"The same results have been documented..."

"The same result has also been reported..."

"The same trends have been reported..."

Use alternatives:

"Similarly,"

"Consistent with previous reports,"

"These findings agree with..."

"Comparable observations have been reported..."

7. Statistical Reporting Issues

The manuscript reports many numerical differences but does not consistently indicate:

p-values

standard errors

significance letters

confidence intervals

Ensure that all figures and tables include:

Mean ± SE (or SD)

Number of replicates (n)

Statistical grouping letters

8. Reference Issues

Some references appear unrelated or weakly connected to the cited statements.

Examples:

Ref. 10 (salicylic acid estimation) is cited in climate-change discussion.

Verify all citations carefully because there may be citation-management errors.

A full reference audit is recommended before resubmission.

Based on the manuscript in its current form, I would suggest a Major Revision before publication.

**********

what does this mean?). If published, this will include your full peer review and any attached files.

If you choose “no”, your identity will remain anonymous but your review may still be made public.

**Do you want your identity to be public for this peer review?**  For information about this choice, including consent withdrawal, please see our Privacy Policy

Reviewer #1: **Yes:**  Naveed Ahmad

Reviewer #2: No

Reviewer #3: No

**********

[NOTE: If reviewer comments were submitted as an attachment file, they will be attached to this email and accessible via the submission site. Please log into your account, locate the manuscript record, and check for the action link "View Attachments". If this link does not appear, there are no attachment files.]

To ensure your figures meet our technical requirements, please review our figure guidelines: https://journals.plos.org/plosone/s/figures

You may also use PLOS’s free figure tool, NAAS, to help you prepare publication quality figures: https://journals.plos.org/plosone/s/figures#loc-tools-for-figure-preparation.

NAAS will assess whether your figures meet our technical requirements by comparing each figure against our figure specifications.

# Author comment: Author response to Decision Letter 1

**DOI:** 10.1371/journal.pone.0356384.r002

16 Jul 2026

Response to Reviewers’ Comments:

PONE-D-26-29102

Endophytic Aspergillus fumigatus Modulates Growth, Oxidative Stress, and Cadmium Accumulation in Okra Under Salinity and Heavy-Metal Stress

PLOS One

Reviewer #1:

This manuscript reports on the isolation of the endophytic fungus Aspergillus fumigatus (strain SOA) from Ziziphus lotus roots and its ability to enhance growth, modulate phytohormone profiles, elevate antioxidant defenses, and reduce cadmium accumulation in okra (Abelmoschus esculentus) under salt and heavy-metal stress conditions. The study addresses a relevant agricultural challenge, employs a reasonably comprehensive experimental design, and presents generally well-supported conclusions. The manuscript is suitable for publication in PLOS ONE following minor revisions as detailed below.

Minor comments:

1. Line 64: “soil salinization is increasing by nearly 10% per year” — this statement requires a citation. Please add the appropriate reference.

Response: Thank you for your valuable suggestion. We have added an appropriate reference to support the statement "soil salinization is increasing by nearly 10% per year" in Line 64 of the revised manuscript.

2. Lines 279–286 (Section 3.4): Metabolite values are reported with varying decimal precision (e.g., 17.64, 0.0769). Please standardize significant figures consistently throughout the Results.

Response: Thank you for your valuable comment. We have carefully reviewed the Results section and standardized the numerical values by using consistent decimal precision/significant figures throughout the manuscript.

3. Line 492: “alt” appears to be a typographical error for “Salt.” Please correct.

Response: Thank you for your careful observation. The typographical error has been corrected, and "alt" has been changed to "Salt".

4. Figure 6 caption refers to measurements over “three weeks” in the text, while figure panels show “1 week,” “2 weeks,” and “7 weeks.” Reconcile caption and figure labels.

Response: Thank you for pointing out this inconsistency. We have carefully reviewed Figure 6 and corrected the discrepancy between the figure caption and the panel labels. The caption has been revised to accurately reflect the time points presented in the figure (1 week, 2 weeks, and 7 weeks), ensuring consistency between the figure and its description throughout the manuscript.

5. Figure 11 legend (line 758) references “IBH + salt stress” — “IBH” appears to be a typographical error; likely intended as “HM” or “HM+Salt.” Please correct.

Response: Thank you for bringing this typographical error to our attention. We have corrected the Figure 11 legend by replacing the incorrect term “IBH + salt stress” with the correct treatment, HM + Salt.

6. Reference 10 (Warrier et al., 2013) is cited in the context of global temperature increase predictions but appears to be a paper on salicylic acid estimation in Eucalyptus. This appears to be a misplaced citation. Please verify and replace with an appropriate climate science reference.

Response: Thank you for pointing out this error. We carefully reviewed the cited reference and confirmed that it was incorrectly cited in the context of global temperature increase predictions. The misplaced citation has been replaced with an appropriate reference that accurately supports the statement.

7. Reference 73 (Zhang et al., 2026) is listed with a publication year of 2026. If this is an in-press or advance online publication, please provide the DOI and indicate “in press” or “advance online publication” as appropriate per PLOS ONE style.

Response: The publication is already online. The page number and DOI has been added to the reference in the list.

8. The abbreviation “FCF” (fungal culture filtrate) used in Section 2.5 (line 178) is not defined at first use. Please define it upon first appearance in the text.

Response: Thank you for your valuable comment. We have defined the abbreviation fungal culture filtrate (FCF) at its first occurrence in Section 2.5 to improve clarity.

9. Section 2.6 lists eight treatments but the Discussion (line 383) refers to “Aspergillus and Trichoderma species” as a comparative context without a corresponding treatment in this study. This reference is fine but could be slightly misleading; consider adding a brief clarifying phrase that this is a literature comparison.

Response: Thank you for your valuable suggestion. Since Aspergillus fumigatus was one of the fungal species used in the present study, we specifically referred to Aspergillus fumigatus rather than the broader genus Aspergillus and added an appropriate supporting reference.

10. The keywords include “zero hunger,” which, while thematically relevant to food security, is an SDG policy term rather than a scientific keyword. Authors may wish to consider replacing it with a more specific scientific term (e.g., “food security,” “crop productivity,” or “sustainable agriculture”) for better indexing.

Response: Thank you for your valuable suggestion. We have replaced zero hunger with "sustainable agriculture," which more accurately reflects the scientific focus of the study. The keyword list has been revised accordingly.

Reviewer #2:

Title: Endophytic Aspergillus fumigatus Modulates Growth, Oxidative Stress, and Cadmium Accumulation in Okra Under Salinity and Heavy-Metal Stress

The manuscript investigates the role of an endophytic fungal strain, Aspergillus fumigatus SOA, in improving okra growth under salinity and cadmium stress. The authors isolated the fungus from Ziziphus lotus roots and evaluated its plant growth-promoting traits, including hormone production, antioxidant response, secondary metabolites, and cadmium modulation. The study reports that SOA improved plant height, biomass, chlorophyll content, and phytohormonal balance under stress conditions. It also suggests that SOA may help reduce oxidative damage and modulate cadmium uptake in okra. Overall, the work addresses a relevant issue in sustainable agriculture and stress physiology.

The topic is timely because salinity and heavy-metal contamination are serious threats to crop productivity. The use of an endophytic fungus as a biological tool for improving okra tolerance is interesting and practically relevant. The manuscript includes a wide range of physiological, biochemical, hormonal, antioxidant, and elemental analyses, which gives the study good experimental coverage. Molecular identification of the fungal isolate through ITS sequencing strengthens the reliability of the work.

1. The abstract is generally clear, but it would benefit from more precise reporting of the experimental conditions. The authors should briefly mention the salt and cadmium stress levels and include one or two key numerical findings beyond plant height.

Response: We sincerely thank the reviewer for this valuable suggestion. The abstract has been revised to provide a clearer description of the experimental conditions by specifying the concentrations of NaCl and Cd used to impose salt and cadmium stress. In addition, we have included key quantitative findings beyond plant height, such as improvements in biomass and chlorophyll content (as appropriate), to better highlight the significance of the study. These revisions provide a more informative and balanced summary of the experimental design and major outcomes.

2. The introduction contains some repetition about abiotic stress, salinity, and physiological damage. The authors should shorten these repeated parts and focus more directly on the specific gap: the limited evidence on endophytic fungi for okra under combined salt and cadmium stress.

Response: Thank you for this valuable suggestion. The Introduction has been carefully revised to improve clarity and avoid repetition. Repeated descriptions of abiotic stress, salinity, and their associated physiological effects have been condensed, resulting in a more concise and focused narrative. Greater emphasis has been placed on the specific research gap, namely the limited evidence regarding the use of endophytic fungi, particularly Aspergillus fumigatus, to enhance okra tolerance under the combined stress of salinity and cadmium.

3. The novelty of the work should be stated more clearly at the end of the introduction. It should be clear whether the novelty lies in the fungal strain, the plant model, the combined stress treatment, or the multi-parameter evaluation.

Response: Thank you for this valuable suggestion. The novelty of the study has been explicitly stated at the end of the Introduction. The revised text clearly highlights that the originality of this work lies in evaluating an endophytic Aspergillus fumigatus strain isolated from Ziziphus lotus for improving okra tolerance under combined salinity and cadmium stress.

4. Some methodological wording needs correction. For example, phrases such as “misleading morphological features,” “chapel medium,” and “duplicate the osmotic pressure” are unclear and should be revised for scientific accuracy.

Response: Thank you for your constructive comment. We have revised this section to improve its scientific clarity and precision.

5. The fungal isolation section is somewhat repetitive. The authors describe PDA sub-culturing and Czapek broth culturing more than once. This section should be streamlined to improve readability.

Response: Thank you for your valuable suggestion. We have carefully revised the fungal isolation section to improve its clarity and readability. Repetitive descriptions of PDA sub-culturing and Czapek broth culturing have been removed.

6. The surface sterilization protocol should be checked. The manuscript mentions 70% ethanol followed by NaCl 2.0%. Usually, sodium hypochlorite is used for surface sterilization, not sodium chloride. If NaCl was actually used, the authors should justify it; otherwise, this should be corrected.

Response: Thank you for your careful observation. The reagent used for surface sterilization was 2% sodium hypochlorite (NaOCl), not 2% sodium chloride (NaCl). This was a typographical mistake in the manuscript. We have corrected the text accordingly to accurately describe the surface sterilization protocol (70% ethanol followed by 2% sodium hypochlorite.

7. Units should be checked throughout the manuscript. For example, GA is reported as mg/mL in fungal profiling, while other hormones are reported in µg/mL, and plant hormones are later reported as mg/g FW. These unit differences should be verified and made consistent.

Response: We have carefully reviewed all units reported throughout the manuscript and corrected any inconsistencies. We confirmed that the units used for fungal metabolite profiling and plant tissue hormone analysis represent different measurement types and have ensured that they are reported appropriately and consistently. Specifically, phytohormone concentrations in fungal culture filtrates are now presented using uniform concentration units (e.g., µg/mL), while endogenous phytohormone levels in plant tissues are consistently expressed on a fresh weight basis (e.g., µg/g FW). In addition, all tables, figures, and the Materials and Methods section were carefully checked to ensure that units are accurate, standardized, and consistent throughout the revised manuscript

8. The discussion is relevant but somewhat repetitive. Several paragraphs restate the results and then compare them with previous studies. The authors should reduce repetition and add a deeper mechanistic explanation of hormone regulation, ROS detoxification, and possible Cd sequestration or immobilization.

Response: We have thoroughly revised the Discussion to improve its scientific depth and readability. Specifically, repetitive descriptions of the experimental results have been removed or substantially condensed, and greater emphasis has been placed on interpreting the underlying physiological and biochemical mechanisms rather than restating the findings.

To strengthen the Discussion, we incorporated a more detailed mechanistic explanation of how endophytic fungal inoculation regulates phytohormone homeostasis under cadmium (Cd) stress. The revised text explains that increased levels of growth-promoting hormones, including indole-3-acetic acid (IAA) and gibberellic acid (GA), together with reduced abscisic acid (ABA) accumulation, contribute to sustained cell division, root development, photosynthetic activity, and stress adaptation under Cd toxicity.

9. The figures and legends require careful proofreading. Treatment labels, units, abbreviations, and spelling should be checked throughout. Any inconsistent terms, such as HM, heavy metal, Cd, salt, NaCl, and SOA, should be standardized.

Response: In response, all figures and figure legends have been thoroughly proofread and revised to improve consistency, accuracy, and clarity. Treatment labels, units of measurement, abbreviations, and spelling have been carefully checked throughout the manuscript.

Specifically, inconsistent terminology has been standardized across all figures, legends, tables, and the main text. The terms "HM" and "heavy metal" have been consistently replaced with "cadmium (Cd)" where appropriate, while "salt" has been revised to the specific treatment "NaCl" only where salinity is discussed and removed where not applicable to avoid confusion. Similarly, the abbreviation "SOA" has been used consistently throughout the manuscript after its first definition, and all other abbreviations have been defined at their first occurrence and applied uniformly thereafter.

Reviewer #3:

1. Inconsistency in Experimental Replication

The manuscript reports (Lines 242–243)"All experiments were performed in three biological replicates"

However, earlier:

"All treatments had six plants..." (Lines 177–184) and "Each replicated six times" (Line 183)

These statements are contradictory.

Number of pots per treatment

Number of plants per pot

Number of biological replicates

Number of technical replicates

Response: The text regarding biological and technical replicates were verified. It has been corrected throughout the manuscript to accurately reflect the experimental design. We have also revised the Materials and Methods section to clearly describe the number of pots, plants per pot, biological replicates, and the replication used for statistical analyses, thereby eliminating any ambiguity and ensuring consistency throughout the manuscript.

2. Units Need Verification

Several hormone concentrations appear unusually high:

Examples:

GA = 20–28 mg/mL

IAA = 24–31 µg/mL

For fungal culture filtrates, GA concentrations in mg/mL are extremely high and likely incorrect.

Check original calculations and units carefully.

Possible intended units:

µg/mL

ng/mL

This requires verification before publication.

Response: We have carefully reviewed all units reported throughout the manuscript and corrected any inconsistencies. We confirmed that the units used for fungal metabolite profiling represent different types of measurements and have ensured that they are reported appropriately and consistently. GA measurement units were verified and corrected.

3. Cadmium Uptake Interpretation

In Results:

Control = 9.979 mg/L Cd

SOA = 12.65 mg/L Cd

HM = 13.65 mg/L Cd

HM+SOA = 10.95 mg/L Cd

The conclusion:

"SOA reduced heavy metal toxicity by modulating cadmium uptake"is only partially supported.

SOA alone increased Cd accumulation compared with control.

Recommended wording:

"SOA reduced Cd accumulation under heavy-metal stress conditions compared with HM treatment alone." instead of a broad statement that SOA always reduces Cd uptake.

Response: The conclusion section were modified and re-written, specifically the Cd accumulation statement, as suggested.

4. Surface Sterilization Procedure

Line 120 it is mentioned "treated with 70% ethanol for 1 minute, NaCl (2.0%) for 2 minutes"

Normally fungal isolation protocols use “Sodium hypochlorite (NaOCl)” not NaCl.

It should be mentioned clearly that whether NaCl was actually used, or NaOCl.

Response: Thank you for your careful observation. The reagent used for surface sterilization was 2% sodium hypochlorite (NaOCl), not 2% sodium chloride (NaCl). This was a typographical mistake in the man

Attachment Submitted filename: Response to editorss and reviewers comments.docx

# Peer review: Decision Letter 1

**DOI:** 10.1371/journal.pone.0356384.r003

**Authors:** Wajid Zaman

3 Aug 2026

Endophytic Aspergillus fumigatus Modulates Growth, Oxidative Stress, and Cadmium Accumulation in Okra Under Salinity and Heavy-Metal Stress

PONE-D-26-29102R1

Dear Dr. Ahmad,

We’re pleased to inform you that your manuscript has been judged scientifically suitable for publication and will be formally accepted for publication once it meets all outstanding technical requirements.

Within one week, you’ll receive an e-mail detailing the required amendments. When these have been addressed, you’ll receive a formal acceptance letter and your manuscript will be scheduled for publication.

An invoice will be generated when your article is formally accepted. Please note, if your institution has a publishing partnership with PLOS and your article meets the relevant criteria, all or part of your publication costs will be covered. Please make sure your user information is up-to-date by logging into Editorial Manager at Editorial Manager® and clicking the ‘Update My Information’ link at the top of the page. For questions related to billing, please contact billing support.

If your institution or institutions have a press office, please notify them about your upcoming paper to help maximize its impact. If they’ll be preparing press materials, please inform our press team as soon as possible -- no later than 48 hours after receiving the formal acceptance. Your manuscript will remain under strict press embargo until 2 pm Eastern Time on the date of publication. For more information, please contact onepress@plos.org.

Kind regards,

Wajid Zaman

Academic Editor

PLOS One

Additional Editor Comments (optional):

Reviewers’ comments:

Reviewer’s Responses to Questions

**Comments to the Author**

Reviewer #1: All comments have been addressed

Reviewer #2: All comments have been addressed

Reviewer #3: All comments have been addressed

**********

2. Is the manuscript technically sound, and do the data support the conclusions?

Reviewer #1: Yes

Reviewer #2: Yes

Reviewer #3: Yes

**********

3. Has the statistical analysis been performed appropriately and rigorously?

Reviewer #1: Yes

Reviewer #2: Yes

Reviewer #3: Yes

**********

4. Have the authors made all data underlying the findings in their manuscript fully available?

The PLOS Data policy

Reviewer #1: Yes

Reviewer #2: Yes

Reviewer #3: Yes

**********

5. Is the manuscript presented in an intelligible fashion and written in standard English?

Reviewer #1: Yes

Reviewer #2: Yes

Reviewer #3: Yes

**********

Reviewer #1: The authors have improved the manuscript thoroughly based on my previous comments and therefore I recommend the acceptance of this work in its current form.

Reviewer #2: fter carefully evaluating the revised manuscript entitled “Endophytic Aspergillus fumigatus Modulates Growth, Oxidative Stress, and Cadmium Accumulation in Okra Under Salinity and Heavy-Metal Stress”, I find that the authors have addressed the reviewers’ comments satisfactorily and have made appropriate revisions that improve the clarity and quality of the manuscript. The study presents valuable findings on the role of an endophytic fungus in enhancing stress tolerance and reducing cadmium accumulation in okra, contributing to the understanding of plant–microbe interactions under combined abiotic stresses. The experimental approach is sound, the data support the conclusions, and the manuscript is now suitable for publication. I recommend acceptance after this first revision, as no major concerns remain. Only minor editorial polishing, if deemed necessary by the editorial office, may be undertaken during the production process.

Reviewer #3: Authors have incorporated all revisions! There are no more quires, which is why I recommend its publication in PLOS ONE.

**********

what does this mean?). If published, this will include your full peer review and any attached files.

If you choose “no”, your identity will remain anonymous but your review may still be made public.

**Do you want your identity to be public for this peer review?**  For information about this choice, including consent withdrawal, please see our Privacy Policy

Reviewer #1: **Yes:**  Naveed Ahmad

Reviewer #2: No

Reviewer #3: No

**********

# Editor report: Acceptance letter

**DOI:** 10.1371/journal.pone.0356384.r004

**Authors:** Wajid Zaman

PONE-D-26-29102R1

PLOS One

Dear Dr. Ahmad,

I’m pleased to inform you that your manuscript has been deemed suitable for publication in PLOS One. Congratulations! Your manuscript is now being handed over to our production team.

At this stage, our production department will prepare your paper for publication. This includes ensuring the following:

* All references, tables, and figures are properly cited

* All relevant supporting information is included in the manuscript submission,

* There are no issues that prevent the paper from being properly typeset

You will receive further instructions from the production team, including instructions on how to review your proof when it is ready. Please keep in mind that we are working through a large volume of accepted articles, so please give us a few days to review your paper and let you know the next and final steps.

Lastly, if your institution or institutions have a press office, please let them know about your upcoming paper now to help maximize its impact. If they’ll be preparing press materials, please inform our press team within the next 48 hours. Your manuscript will remain under strict press embargo until 2 pm Eastern Time on the date of publication. For more information, please contact onepress@plos.org.

You will receive an invoice from PLOS for your publication fee after your manuscript has reached the completed accept phase. If you receive an email requesting payment before acceptance or for any other service, this may be a phishing scheme. Learn how to identify phishing emails and protect your accounts at https://explore.plos.org/phishing.

If we can help with anything else, please email us at customercare@plos.org.

Thank you for submitting your work to PLOS ONE and supporting open access.

Kind regards,

PLOS ONE Editorial Office Staff

on behalf of

Dr. Wajid Zaman

Academic Editor

PLOS One
